# Nociceptor neurons suppress antitumor immunity in breast cancer

**DOI:** 10.21203/rs.3.rs-9927184/v1

**Published:** 2026-06-09

**Authors:** Yue Wu, Maryam Ahmadi, Ania Bogoslowski, Laura Brabenec, Jumana Abbadi, Beatriz G. S. Rocha, Amin Reza Nikpoor, Vera Thiel, Elisa Preto, Sheu O. Sulaiman, Migmar Tsamchoe, Olga Bednova, Alissa Dory, Lisa A. McIlvried, Tuany Eichwald, Tamara McErlain, Mohammad Balood, Christos Boutopoulos, Moutih Rafei, Paola Vermeer, Aeson Chang, Erica Sloan, Moran Amit, Josef Penninger, H. Uri Saragovi, Andreas Trumpp, Nicole N. Scheff, Karen Dixon, Maureen Cox, Alexander Birbrair, Sébastien Talbot, Jeremy Borniger

**Affiliations:** 1 Cold Spring Harbor Laboratory, Cold Spring Harbor, NY 11724, USA; 2 Department of Biomedical and Molecular Sciences, Queen’s University, Kingston, ON, Canada; 3 Department of Medical Genetics, Life Sciences Institute, University of British Columbia, Vancouver, BC, Canada; 4 Department of Physiology and Pharmacology, Karolinska Institutet, Solna, Sweden; 5 Department of Microbiology and Immunology, University of Oklahoma Health Sciences Center, Oklahoma City, OK, USA; 6 Department of Molecular Biology, Adelson School of Medicine, Ariel University, Ariel, Israel; 7 Heidelberg Institute for Stem Cell Technology and Experimental Medicine (HI-STEM), Heidelberg University, Heidelberg, Germany; 8 Department of Biomedicine, University of Basel, Basel, Switzerland; 9 Institute of Biological Sciences, Federal University of Minas Gerais, Brazil; 10 Lady Davis Institute, Jewish General Hospital, McGill University, Centre for Translational Research, Montreal, QC, Canada; 11 Department of Pharmacology and Therapeutics, McGill University, Montreal, QC, Canada; 12 Centre de recherche de l’Hôpital Maisonneuve-Rosemont, Montréal, QC, Canada; 13 Hillman Cancer Center, Department of Neurobiology, University of Pittsburgh, Pittsburgh, PA, USA; 14 Department of Pharmacology and Physiology, Université de Montréal, Montreal, QC, Canada; 15 Cancer Biology and Immunotherapies Group, Sanford Research, Sioux Falls, SD, USA; 16 Monash Institute of Pharmaceutical Sciences, Monash University, Parkville, VIC, Australia; 17 Department of Head and Neck Surgery, The University of Texas MD Anderson Cancer Center, Houston, TX, USA; 18 Department of Dermatology, University of Wisconsin–Madison, Madison, WI, USA

## Abstract

Peripheral nerves are emerging regulators of the tumor microenvironment, but how sensory innervation shapes breast cancer immunity remains poorly defined. Here we show that triple-negative breast cancers (TNBCs) co-opt nociceptor neurons to suppress antitumor immunity and promote disease progression. Across orthotopic TNBC models, we found that primary tumors and tumor-draining lymph nodes were densely innervated by CGRP^+^ sensory fibers. Tumor-derived cues directly activated dorsal root ganglion neurons, increased calcium responsiveness, induced Ngfr and Atf3, and triggered release of CGRP and substance P. Mechanistically, a tumor-derived proNGF-NGFR axis reprogrammed nociceptors and promoted neuropeptide secretion. Soluble mediators from activated nociceptors suppressed CD8^+^ T cell-mediated tumor-cell killing, whereas sensory-neuron silencing or ablation curtailed tumor growth and remodeled the immune microenvironment toward dendritic-cell activation, myeloid reprogramming, and enhanced CD8^+^ T cell and NK-cell effector states. Subset-specific analysis revealed nonredundant sensory control of immune states, with MrgD^+^ neurons selectively shaping macrophage-centered programs. Finally, blockade of CGRP signaling through RAMP1 reduced tumor growth and markedly enhanced PD-1 blockade, nearly eliminating primary tumor burden and lung metastasis in vivo. T cell-specific Ramp1 deletion similarly restrained tumor growth, and RAMP1^+^ CD8^+^ T cells in human TNBC displayed an exhaustion-associated phenotype. Together, these findings define a tumor-promoting proNGF-nociceptor-CGRP-RAMP1 axis and identify neuroimmune signaling as a therapeutically actionable vulnerability in TNBC.

## INTRODUCTION

Cancer neuroscience has reframed solid tumors as aberrantly innervated ecosystems in which malignant, stromal, immune and neural cells exchange signals that shape disease progression^1-3^. This framework is especially relevant to breast cancer, where durable immune control remains difficult despite advances in immunotherapy. Immune-checkpoint blockade has improved outcomes for a subset of patients with TNBC^4-7^, yet many tumors maintain a microenvironment in which CD8^+^ T cells lose effector function, upregulate inhibitory receptors such as PD-1, CTLA-4, LAG-3 and TIM-3, and fail to sustain cytotoxic activity^8-10^. This dysfunctional state is reinforced by suppressive myeloid cells, abnormal vasculature and stromal barriers^11-15^. Most efforts to reverse immune escape have therefore focused on tumor-intrinsic oncogenic programs or immune-cell-intrinsic checkpoints. The peripheral nervous system is emerging as an additional, incompletely understood component of this landscape, with roles in angiogenesis, metastasis, stress adaptation and antitumor immunity.

Nociceptor neurons are particularly well positioned to influence tumor immunity. These specialized sensory neurons detect tissue damage and noxious stimuli, but they also function as immunologically competent sentinel cells^16-19^. They express receptors for cytokines, chemokines, acidosis, neurotrophins and damage-associated signals, enabling direct detection of inflammatory and neoplastic tissue states^20-22^. Upon activation, nociceptors release neuropeptides, including calcitonin gene-related peptide (CGRP) and substance P; alter vascular permeability; and shape the behavior of macrophages, dendritic cells, innate lymphoid cells and T cells^23,24^. Studies in infection and inflammatory disease show that these neuroimmune circuits are highly context dependent: nociceptors can either amplify or restrain immunity depending on tissue site, timing, neuronal subtype and the responding immune population^25,26^. These principles raise the possibility that tumors hijack nociceptor programs to establish immune-permissive niches.

Consistent with this idea, increased nerve density has been described in many solid tumors, and tumors can actively recruit or expand neural networks through neurotrophin-driven axonogenesis-like programs, including pathways involving nerve growth factor (NGF)^27,28^. Cancer-derived extracellular vesicles may further amplify this process^29,30^. Studies in melanoma, pancreatic cancer, head and neck cancer and other malignancies have shown that sensory neurons are active participants in tumor progression rather than passive bystanders^17,31-35^. In these settings, nociceptor-derived neuropeptides can promote tumor growth, reshape stromal programs, suppress NK-cell function and drive T cell dysfunction. CGRP signaling through the RAMP1-CALCRL receptor complex has emerged as a candidate pathway linking nociceptor activity to immune suppression^35,36^ , whereas NGF-dependent activation or expansion of CGRP^+^ fibers may provide an upstream neurotrophic framework^37,38^. Whether an analogous nociceptor-neuropeptide circuit operates in breast cancer, and which sensory subsets and immune pathways it controls, remains unclear.

Resolving these questions is important because sensory neurons are anatomically positioned to influence both local tumor biology and regional antitumor immunity. Nociceptors innervate peripheral tissues and can access tumor-draining lymph nodes, raising the possibility that they regulate effector responses within tumors as well as the priming, trafficking or maintenance of cytotoxic lymphocytes^39^. Dorsal root ganglion neurons are also heterogeneous, comprising peptidergic CGRP^+^ subsets, TRPV1^+^ nociceptors and nonpeptidergic MrgD^+^ neurons^16,40^. Distinct sensory circuits may therefore exert nonredundant effects on myeloid and lymphoid compartments. Defining these circuits is essential for understanding how breast tumors exploit the peripheral nervous system and for identifying therapeutic nodes that can be targeted without broadly disrupting beneficial neural functions.

Here, we define a nociceptor-centered neuroimmune circuit that promotes breast tumor progression. Using orthotopic TNBC models, neuronal tracing, calcium imaging, co-culture systems, transcriptional profiling, chemogenetic activation, genetic ablation and pharmacological intervention, we show that breast tumors and their draining lymph nodes are densely innervated by CGRP^+^ nociceptors. We identify tumor-derived proNGF-NGFR signaling as an upstream mechanism that directly activates and reprograms sensory neurons, increasing neuronal stress responses and neuropeptide release. Functionally, nociceptor-derived soluble mediators suppress CD8^+^ T cell cytotoxicity, whereas sensory-neuron ablation restrains tumor growth and shifts the immune microenvironment toward dendritic-cell activation, myeloid remodeling, and stronger CD8^+^ T cell and NK-cell responses. Finally, we identify CGRP-RAMP1 signaling as a downstream pathway through which nociceptors limit antitumor immunity and responsiveness to checkpoint blockade. Together, these findings position tumor-innervating nociceptors as active regulators of breast cancer immunity and nominate neuroimmune signaling as a tractable therapeutic vulnerability in TNBC.

## RESULTS

### CGRP^+^ nociceptors innervate breast tumors and tumor-draining lymph nodes

**Figure 1.**
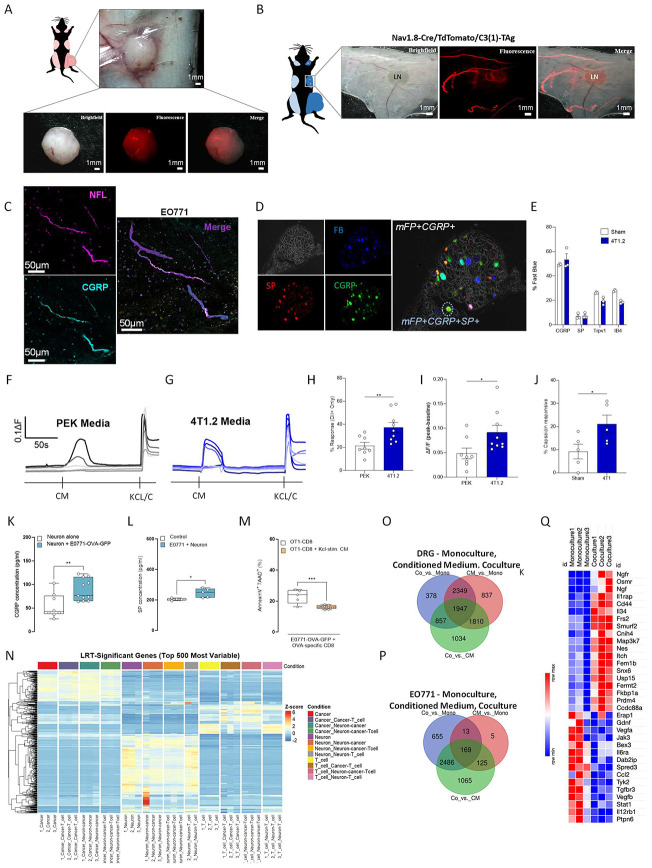
Sensory innervation and neuroimmune crosstalk in breast tumors A,B, Mammary fat pads from 32-week-old female C3(1)-TAg/Nav1.8-^Cre^::tdTomato^fl/wt^ nociceptor reporter mice with breast cancer were harvested, and innervation of the primary tumor (A) and draining lymph node (B) was assessed by brightfield and fluorescence microscopy. Dense peritumoral and perilymphatic fibers indicate that TNBCs and their tumor-draining lymph nodes are richly innervated by nociceptor neurons. Scale bars, 1 mm. C, EO771 TNBC cells (2.5 × 10^5^) were orthotopically injected into the fourth mammary fat pad of 8-week-old female C57BL/6 wild-type mice. Tumors were harvested on day 10, and innervation subtypes were characterized by fluorescence microscopy. CGRP^+^ fibers are shown in turquoise and NFL fibers in magenta. Scale bars, 50 μm. D,E, 4T1.2 TNBC cells (1 × 10^6^) were orthotopically injected into the fourth mammary fat pad of 8-week-old female BALB/c mice. One week before tumor implantation, the mammary fat pad was injected with the retrograde tracer Fast Blue. T10-T13 DRGs, which contain most TNBC-innervating neurons, were harvested on days 24-27. Tumor-innervating neuronal subtypes were characterized (D) and quantified (E) by fluorescence microscopy. Approximately 50% of traced neurons were CGRP^+^, approximately 10% were substance P^+^ and approximately 20% were IB4^+^ nonpeptidergic neurons, indicating that tumor-innervating neurons are predominantly CGRP^+^ nociceptors. F-J, Three weeks before calcium-imaging experiments, the fourth mammary fat pad of 8-week-old BALB/c mice was injected with the retrograde tracer DiI. Conditioned media were collected from PEK control cells and 4T1.2 cells cultured for 48 h at 80-90% confluence. In parallel, T10-T13 DRGs from naive 8-week-old female BALB/c mice were dissociated and recorded within 6 h. Neurons were loaded with Fura-2AM and exposed to conditioned medium for 60 s, followed by KCl (30 mM; 4 s) and capsaicin (500 nM; 4 s). Calcium flux was recorded in small-diameter neurons (<40 μm). Exposure to 4T1.2 conditioned media increased neuronal responsiveness (H), response amplitude (I) and the proportion of capsaicin-responsive neurons (J) relative to PEK conditioned media, indicating activation and sensitization of nociceptors by tumor-derived soluble factors. K,L, T7-T12 DRGs from naive 8-week-old female C57BL/6 mice were cultured alone or co-cultured with EO771-OVA-GFP cells at a 1:10 ratio for 72 h. In the presence of peptidase and protease inhibitors, conditioned medium was collected and spontaneous neuropeptide release was quantified by ELISA. Compared with neurons cultured alone, co-cultured neurons released more CGRP (K) and substance P (L), indicating direct activation of DRG neurons by cancer cells. [AUTHOR QUERY: add statistical test and n values.] M, T7-T12 DRGs from naive 8-week-old female BALB/c mice were cultured for 24 h and then stimulated with KCl (50 mM). Conditioned medium was collected after 30 min. In parallel, EO771-OVA-GFP cells were co-cultured at a 1:10 ratio with magnetically purified splenic CD8^+^ T cells from naive 8-week-old female OT-I mice. These co-cultures were exposed, or not, to KCl-induced neuronal conditioned medium for 24 h (100 μL collected from 500 μL in a 96-well plate containing 10,000 neurons), and elimination of EO771-OVA-GFP cells was quantified by flow cytometry. Neuronal conditioned medium reduced CD8^+^ T cell cytotoxicity by approximately 50%, indicating that nociceptor-derived soluble factors exert a strong immunosuppressive effect. N, Naive DRG neurons (Trpv1-Cre::tdTomato^fl/wt), EO771-OVA-GFP cells and OVA-specific cytotoxic CD8^+^ T cells were cultured alone or in combination. After 48 h, cells were collected, FACS-purified and subjected to RNA sequencing. Hierarchical clustering of sorted transcriptional profiles revealed distinct condition-specific gene modules, consistent with extensive reprogramming across mono-, co- and tri-culture conditions. O-Q, Naive DRG neurons were cultured alone, co-cultured with EO771-GFP cells, or exposed to tumor-conditioned medium (O). In reciprocal experiments, EO771-GFP cells were cultured alone, co-cultured with naive DRG neurons, or exposed to DRG neuron-conditioned medium (P). After 48 h, cells were collected, FACS-purified and subjected to bulk RNA sequencing. Hierarchical clustering of sorted neuronal transcriptional profiles revealed distinct condition-specific gene clusters, including increased Ngfr expression (Q), suggesting that tumor cells directly reprogram nociceptors and alter their capacity to sense growth-factor cues.

We first asked whether TNBC lesions acquire sensory innervation. To test this, we generated C3(1)-TAg;Nav1.8-tdT nociceptor-reporter mice, which spontaneously develop breast tumors. At 32 weeks of age, tdTomato^+^ nerve fibers were abundant in both primary tumors and tumor-draining lymph nodes, indicating that TNBC establishes sensory innervation at local and regional disease sites (Fig. 1A,B). Human breast cancer biopsies showed variable innervation, with a trend toward higher fiber density in more advanced cases (Supplementary Fig. 1A). In EO771 tumors, CGRP^+^ fibers extensively overlapped with neurofilament light (NFL)^+^ fibers (Fig. 1C). Retrograde tracing from orthotopic 4T1.2 tumors to T10–L2 dorsal root ganglia (DRGs) further showed that tumor-projecting sensory neurons were predominantly CGRP^+^, with smaller fractions expressing substance P or binding IB4 (Fig. 1D,E). Complementary imaging of MMTV-PyMT and 4T1 tumors confirmed the presence of CGRP^+^ fibers associated with neurofilament-labeled structures across breast tumor models (Supplementary Fig. 1B,C). Together, these data identify peptidergic nociceptors as a prominent sensory neuronal population within breast tumors and their draining lymph nodes.

### TNBC-derived cues activate nociceptors and suppress CD8^+^ T cell cytotoxicity

We next asked whether tumor-derived soluble factors directly activate sensory neurons. Conditioned medium from 4T1.2 cells increased calcium responses in small-diameter DRG neurons, increased the magnitude of neuronal calcium flux and expanded the proportion of capsaicin-responsive neurons, indicating preferential activation and sensitization of nociceptors by tumor-derived cues (Fig. 1F-J). Consistent with direct neuronal activation, co-culture of DRG neurons with EO771-OVA-GFP cells increased spontaneous release of CGRP and substance P into the culture supernatant (Fig. 1K,L). Functionally, conditioned medium from depolarized DRG neurons reduced OT-I CD8^+^ T cell-mediated killing of EO771-OVA-GFP cells by approximately 50%, demonstrating that neuron-derived soluble factors can suppress antitumor cytotoxicity (Fig. 1M).

Tumor-derived cues also promoted structural neuronal remodeling. DRG neurons co-cultured with tumor spheroids extended longer neurites than neurons co-cultured with control spheroids and increasing NGF concentrations or cancer-cell conditioned medium supported progressive neurite extension in time-course assays (Supplementary Fig. 1D,E). These findings support bidirectional crosstalk in which breast cancer cells activate and remodel sensory neurons, while sensory neuron-containing cultures provide soluble signals that alter tumor-immune interactions.

To define the transcriptional consequences of neuron-tumor-immune crosstalk, we performed RNA sequencing after mono-, co- and tri-culture of DRG neurons, tumor cells and OVA-specific cytotoxic CD8^+^ T cells. Each culture condition formed a distinct transcriptional cluster, consistent with broad reprogramming across the interacting cell populations (Fig. 1N). In reciprocal co-culture experiments, purified neurons exposed to tumor cells or tumor-conditioned medium adopted distinct transcriptional states, including increased Ngfr expression, indicating that tumor cells directly reprogram nociceptors and alter their sensitivity to growth-factor cues (Fig. 1O-Q).

### A tumor-derived proNGF-NGFR axis reprograms nociceptors and promotes CGRP release

**Figure 2.**
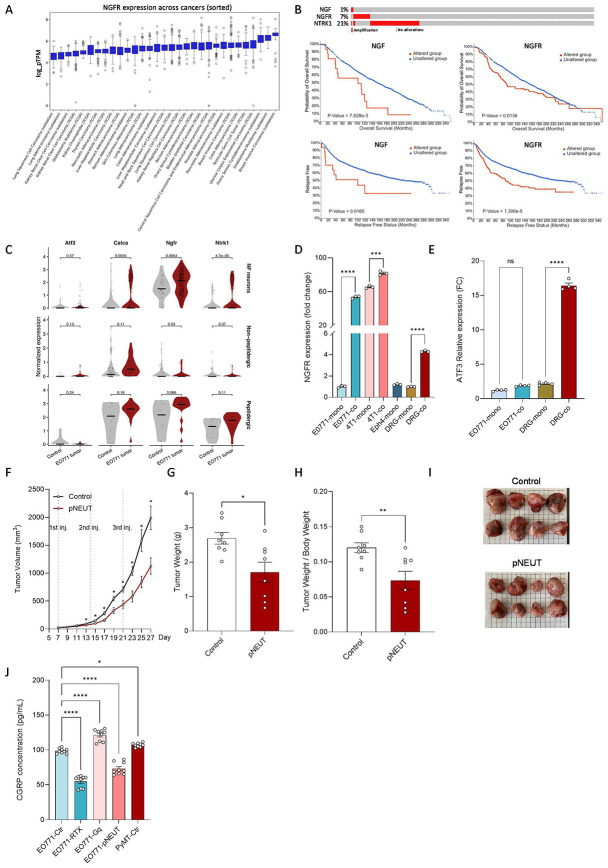
A tumor-derived NGF pathway reprograms nociceptor neurons A, Boxplots of log2-transformed TPM values showing NGFR mRNA expression across cancer types in TCGA and an independent validation cohort. NGFR expression is among the highest in breast invasive carcinoma in TCGA and is high in the validation cohort. B, Analysis of genetic alterations in the NGF-TrkA/p75NTR axis using the METABRIC breast cancer cohort showed that NTRK1 had the highest alteration rate (21%), followed by NGFR (7%) and NGF (1%), with DNA copy-number amplification as the predominant alteration type. NGF alteration was significantly associated with poorer overall survival (p < 0.001) and relapse-free survival (p = 0.016). NGFR alteration was significantly associated with poorer relapse-free survival (p < 0.001) and overall survival (p = 0.0136), whereas NTRK1 alterations were not. These data suggest frequent dysregulation of NGF-pathway genes in breast cancer and implicate NGFR alteration in adverse prognosis. C, 4T1.2 TNBC cells (1 × 10^6^) were orthotopically injected into the fourth mammary fat pad of 8-week-old female BALB/c mice. One week before tumor implantation, the mammary fat pad was injected with the retrograde tracer Fast Blue. T7-T12 DRGs, which contain most TNBC-innervating neurons, were harvested on day 14. Tumor-innervating neurons were then FACS-purified (Fast Blue^+^, tdTomato^+^) and analyzed by single-cell RNA sequencing. Ntrk1 was upregulated in nonpeptidergic neurons, whereas Ngfr was enriched in peptidergic nociceptors, suggesting in vivo reprogramming of nociceptor growth-factor sensing by tumor cells. D,E, T7-T12 DRGs from naive 1-week-old female C57BL/6 mice were cultured for 3 days. In parallel, EO771 and 4T1 TNBC cells were cultured alone or co-cultured with primary DRG neurons. Co-cultured DRG neurons and TNBC cells were then FACS-purified, and Ngfr (D) and Atf3 (E) expression was quantified by qPCR. DRG monoculture and non-malignant Eph4 mammary epithelial cells served as controls. F-I, Eight-week-old C57BL/6 mice bearing orthotopic EO771 tumors were treated with saline control or pNEUT antibody (75 μg per injection) every 7 days (days 7, 14 and 21 after tumor-cell implantation; n = 8 per group). Tumor volumes were measured every other day for 27 days. Tumor growth curves (F), endpoint tumor weight (G), tumor weight normalized to body weight (H) and representative endpoint tumors (I) are shown. pNEUT treatment significantly reduced tumor volume and weight, with a more pronounced reduction after normalization to body weight. Data are presented as mean ± SEM. Statistical significance was determined by an unpaired t-test. J, Naive 4-week-old female C57BL/6 mice were treated with or without resiniferatoxin (RTX) to ablate nociceptors. Four weeks later, mice were orthotopically injected with EO771 breast cancer cells. In parallel, Nav1.8-Cre; hM3Dq^fl/+ mice bearing EO771 tumors received daily intraperitoneal injections of deschloroclozapine (DCZ; 3 μg/kg) to chemogenetically activate Nav1.8^+^ nociceptors. Where indicated, tumor-bearing mice were treated with anti-proNGF antibody on days 7, 14 and 21 after injection. At endpoint, orthotopic tumors were harvested and randomly allocated for tissue processing. CGRP was extracted from tumor tissue lysates and quantified by ELISA. An independent orthotopic PyMT breast tumor model (PyMT-Ctr) was included as a baseline comparison.

Because the co-culture experiments implicated altered neuronal growth-factor sensing, we interrogated public breast cancer datasets for components of the NGF pathway. Across TCGA cancer types, NGFR expression was among the highest in breast invasive carcinoma; an independent validation cohort similarly showed high NGFR expression in breast cancer (Fig. 2A). In the METABRIC breast cancer cohort, alterations in NTRK1, NGFR and NGF were detected in 21%, 7% and 1% of tumors, respectively, with DNA copy-number amplification representing the predominant alteration type. Notably, alterations in NGFR and NGF, but not NTRK1, were associated with significantly worse relapse-free and overall survival, linking dysregulation of this pathway to poor clinical outcome (Fig. 2B; Supplementary Fig. 2A,B).

To determine whether tumor-innervating neurons engage this pathway in vivo, we isolated retrogradely traced tumor-projecting neurons from 4T1.2-bearing mice and profiled them by single-cell RNA sequencing. This analysis revealed subtype-specific remodeling of growth-factor receptor expression, with Ntrk1 upregulated in nonpeptidergic neurons and Ngfr enriched in peptidergic nociceptors (Fig. 2C). In vitro, co-culture with multiple TNBC cell lines increased neuronal Ngfr and Atf3 expression, and proNGF neutralization prevented tumor-induced Ngfr upregulation, identifying tumor-derived proNGF as a driver of neuronal reprogramming (Fig. 2D,E). In EO771-bearing mice, proNGF neutralization reduced tumor growth, endpoint tumor weight and tumor weight normalized to body weight (Fig. 2F-I). Orthotopic EO771 and PyMT tumors exhibited comparable baseline CGRP release from DRG neurons. CGRP levels increased after chemogenetic activation of Nav1.8^+^ nociceptors in Nav1.8-hM3Dq mice (EO771-Gq), but this effect was absent after nociceptor ablation (EO771-RTX) and significantly reduced by anti-proNGF treatment (EO771-αNGF) (Fig. 2J). Together, these findings identify a tumor-derived proNGF-NGFR axis that reprograms nociceptors and promotes neuropeptide release.

### Nociceptor activity promotes breast tumor progression

**Figure 3.**
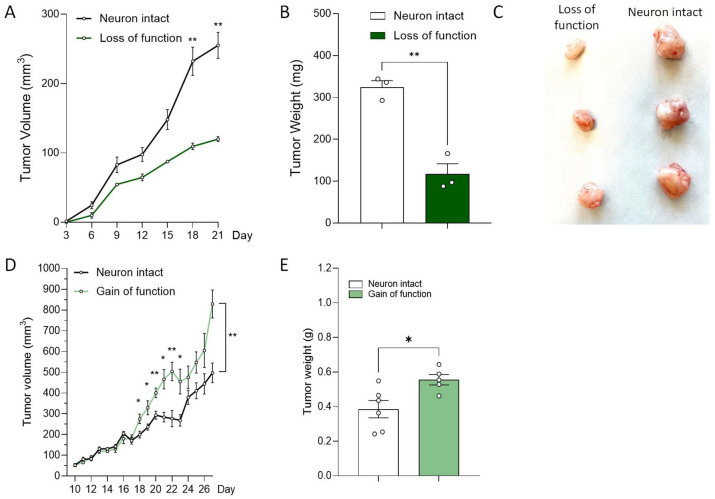
Nociceptor neurons regulate breast tumor progression A-C, Naive 8-week-old female C57BL/6 mice (n = 3) were treated with capsaicin (100 μM, 100 μL) or vehicle for 3 consecutive days to chemoablate nociceptors. Three days later, EO771 TNBC cells (2.5 × 10^5^) were injected into the fourth mammary fat pad. Tumor growth was monitored for 21 days. At endpoint, nociceptor-ablated mice showed reduced tumor growth (A), tumor weight (B) and tumor size (C). D,E, EO771 TNBC cells (1 × 10^6^) were injected into the fourth mammary fat pad of 8-week-old female Nav1.8-Cre::hM3Dq^fl/wt mice, in which nociceptor activity can be controlled chemogenetically. Mice were treated once daily with the hM3Dq-selective agonist CNO (1 mg/kg, i.p.), and tumor growth was monitored for 26 days. At endpoint, chemogenetic activation of nociceptors increased tumor growth (D) and tumor weight (E).

Having established that tumor-derived cues activate and reprogram nociceptors, we next tested whether nociceptors functionally regulate tumor growth in vivo. High-dose capsaicin-mediated nociceptor chemoablation before tumor implantation significantly slowed orthotopic EO771 tumor growth and reduced endpoint tumor weight and tumor size, indicating that nociceptors are required for maximal tumor progression (Fig. 3A-C). Conversely, chemogenetic activation of Nav1.8^+^ nociceptors in Nav1.8-Cre::hM3Dq DREADD mice accelerated tumor growth and increased tumor weight, demonstrating that enhanced nociceptor activity is sufficient to promote tumor progression (Fig. 3D,E). Behavioral assays independently confirmed increased sensory neuron activity in gain-of-function mice, as shown by reduced tail-flick latency and decreased mechanical withdrawal thresholds in the Randall-Selitto assay (Supplementary Fig. 3A,B).

**Figure 4.**
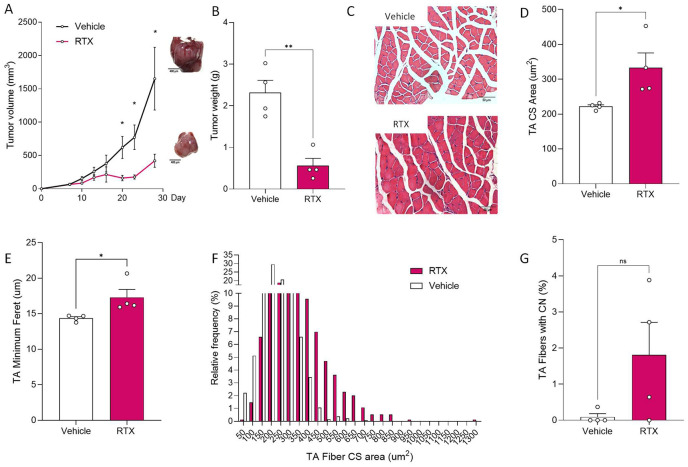
RTX-mediated sensory neuron ablation reduces tumor growth and promotes skeletal muscle remodeling A-G, Naive 4-week-old female C57BL/6 mice were treated with vehicle or RTX on three consecutive days (30, 70 and 100 μg/kg body weight) to chemoablate nociceptors. Four weeks later, EO771 cells (1 × 10^6^) were inoculated into the fourth mammary fat pad. Tumor growth was monitored every 2 days, and muscle wasting was assessed at endpoint. Mice were euthanized 28 days after tumor implantation. Sensory neuron ablation reduced tumor growth (A) and tumor weight (B). It also increased tibialis anterior (TA) myofiber cross-sectional area (C,D) and minimum Feret diameter (E), with fiber-size distribution shifting toward larger fibers (F). Sensory neuron depletion also increased the percentage of TA fibers containing centrally located nuclei (G). These data support a role for nociceptors in promoting tumor growth and tumor-associated muscle remodeling. Groups were compared using an unpaired t-test or a Mann-Whitney test when normality assumptions were not met. n = 4. Data are presented as mean ± SEM. *p < 0.05, **p < 0.01.

Pharmacological inhibition of sensory neuron activity produced the opposite effect. In EO771 tumor-bearing mice, local peritumoral treatment with QX-314, a membrane-impermeant sodium-channel blocker^17,41-44^, or botulinum toxin reduced tumor growth and decreased endpoint tumor weight relative to vehicle-treated controls (Supplementary Fig. 3C,D). These effects were accompanied by improved CD8^+^ T cell effector phenotypes, including fewer PD-1^+^ CD8^+^ T cells after botulinum toxin treatment and increased IFNγ^+^, TNFα^+^ and IFNγ^+^TNFα^+^ CD8^+^ T cell populations after QX-314 and/or botulinum toxin treatment (Supplementary Fig. 3E-G). Similar tumor-suppressive effects were observed in the 4T1 model, where QX-314 reduced tumor growth and endpoint tumor weight (Supplementary Fig. 3H,I). QX-314 treatment also reduced PD-1^+^ and PD-1^+^LAG3^+^ exhausted CD8^+^ T cells while increasing TNFα^+^ and IL-2^+^ CD8^+^ T cells, further supporting a role for sensory neuron activity in limiting antitumor T cell function (Supplementary Fig. 3J-M). Using an independent sensory-ablation strategy, RTX treatment likewise suppressed EO771 tumor growth and reduced tumor weight (Fig. 4A,B). RTX also reduced tumor growth in the 4T1.2 TNBC model (Supplementary Fig. 4), supporting a conserved role for sensory nerves in promoting tumor progression.

Beyond reducing tumor burden, RTX-mediated sensory ablation altered skeletal muscle wasting, a key co-morbid feature of cancer progression^45,46^. Tibialis anterior myofibers from RTX-treated tumor-bearing mice showed increased cross-sectional area and minimum Feret diameter, together with a rightward shift in fiber-size distribution toward larger fibers (Fig. 4C-F). RTX treatment also increased the proportion of fibers containing centrally located nuclei, consistent with altered muscle remodeling (Fig. 4G). Additional muscle analyses showed that sensory nerve depletion increased fiber area and minimum Feret diameter in extensor digitorum longus and gastrocnemius muscle, but not in soleus muscle, indicating muscle-specific effects of sensory denervation on myofiber morphology (Supplementary Fig. 5A-L). These findings suggest that nociceptors contribute not only to primary tumor growth but also to tumor-associated muscle remodeling.

### Global nociceptor ablation enhances antitumor immunity and limits T cell dysfunction

**Figure 5.**
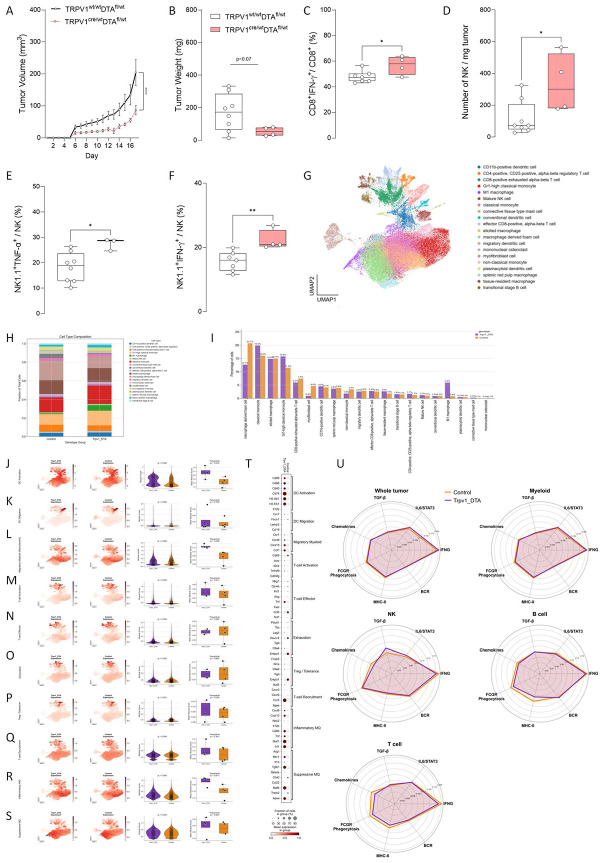
Sensory neuron ablation suppresses tumor growth and enhances antitumor immunity A-F, EO771 cells (2 × 10^5^) were injected into the fourth mammary fat pad of 8-week-old female nociceptor-intact (Trpv1^wt::DTA^fl/wt) and nociceptor-ablated (Trpv1-Cre::DTA^fl/wt) mice. Tumor growth was monitored daily for 16 days, and antitumor immunity was assessed at endpoint by tumor immunophenotyping and single-cell RNA sequencing. Genetic ablation of nociceptor neurons reduced tumor growth (A) and tumor weight (B) and enhanced antitumor immunity, as shown by an increased proportion of IFNγ^+^ CD8^+^ T cells (C), increased tumor infiltration by NK cells (D), and higher frequencies of IFNγ^+^ (E) and TNFα^+^ (F) NK cells. G-I, Single-cell RNA sequencing of tumor-infiltrating immune cells is shown as a t-SNE projection (G). Cell-composition analyses showed that nociceptor ablation increased the proportion of pro-inflammatory macrophages, defined by expression of Cxcl9, Ccl19, Cd86 and Tnfa, while reducing the abundance of unpolarized tissue-resident macrophages and Treg cells (H,I). J-U, Pathway analysis of differentially expressed genes was performed across tumor-infiltrating immune cell subsets. Using a pseudobulk analytical approach, nociceptor ablation increased gene programs associated with dendritic-cell activation (J), dendritic-cell migration (K), T cell responses (L), T cell effector function (M) and M1-like antitumor immunity (P), while reducing exhaustion (N), tolerance (O) and M1-like pro-tumor signatures (Q). At the individual-gene level, tumor-infiltrating CD8^+^ T cells from nociceptor-ablated mice showed reduced expression of the exhaustion markers Havcr2, Pdcd1 and Tox (R).

To define how nociceptors shape the tumor immune microenvironment, we orthotopically implanted EO771 cells into nociceptor-intact and genetically nociceptor-ablated mice. Loss of TRPV1-lineage nociceptors significantly reduced tumor growth and tumor weight (Fig. 5A,B). This effect was accompanied by enhanced antitumor immunity, including an increased proportion of IFNγ^+^ CD8^+^ T cells, greater NK-cell infiltration, and increased frequencies of IFNγ^+^ and TNFα^+^ NK cells within the tumor (Fig. 5C-F). Consistently, in an independent AT-3 orthotopic tumor model, TRPV1-Cre × DTA mice exhibited significantly reduced tumor growth and prolonged survival after tumor implantation (Supplementary Fig. 6A,B).

Single-cell RNA sequencing of tumor-infiltrating leukocytes revealed only modest differences in global low-dimensional clustering between nociceptor-intact and nociceptor-ablated tumors; however, compositional and cell-state analyses showed marked immune remodeling after nociceptor ablation. Tumors from nociceptor-ablated mice contained a higher proportion of pro-inflammatory macrophages expressing *Cxcl9, Ccl19, Cd86* and *Tnfa*, together with fewer unpolarized tissue-resident macrophages and fewer Treg cells (Fig. 5G-I). Pseudobulk pathway analysis further showed that nociceptor ablation increased programs associated with dendritic-cell activation and migration, T cell responses, T cell effector function, and M1-like antitumor immunity, while decreasing exhaustion and tolerance signatures (Fig. 5J-P). Consistent with this shift, tumor-infiltrating CD8^+^ T cells from nociceptor-ablated mice expressed lower levels of the exhaustion-associated genes *Havcr2, Pdcd1* and *Tox*.

Cell-cell communication analysis further supported immune rewiring after TRPV1-lineage neuron ablation. CellChat^47^ analysis revealed genotype-specific changes in incoming signaling strength across tumor-infiltrating immune populations, with prominent signaling activity in tumor-associated macrophages (TAMs) (Supplementary Fig. 6C). Chord plots and pathway-level heatmaps showed altered ligand-receptor communication networks between nociceptor-intact and nociceptor-ablated tumors, including increased signaling through immunoregulatory and inflammatory pathways in nociceptor-ablated tumors (Supplementary Fig. 6D,E). Selected pathway analysis further highlighted enhanced TAM-TAM interactions and altered signaling through mediators including *Cd80, Tgfb1, Tnfrsf1a* and *Tnfrsf1b* following sensory neuron ablation (Supplementary Fig. 6H). Together, these data indicate that nociceptors restrain antitumor immunity by suppressing myeloid activation and favoring dysfunctional T cell states.

### MrgD^+^ sensory neurons selectively regulate macrophage-centered immune programs

**Figure 6.**
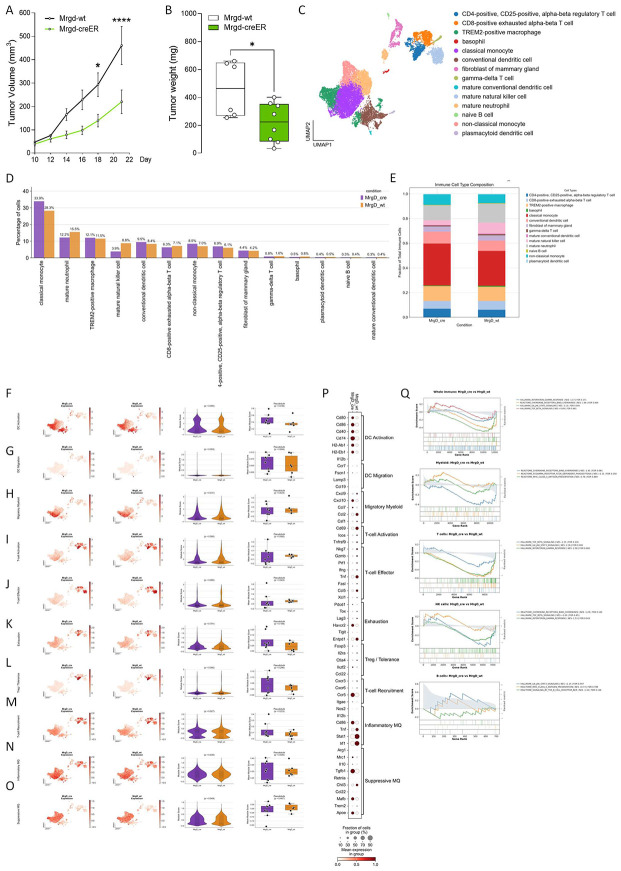
MrgD^+^ sensory neuron ablation suppresses tumor growth and reprograms macrophage-centered immune responses A,B, Eight-week-old female MrgD-CreER^wt::DTA^fl/wt mice and littermate controls (MrgD^wt/wt::DTA^fl/wt) received tamoxifen ([AUTHOR QUERY: add dose and schedule]) in corn oil to induce sensory neuron ablation. EO771-OVA cells (2 × 10^5^ in 50 μL PBS) were injected into the mammary fat pad 28 days after the first tamoxifen administration. Tumor growth (A) was monitored daily for 22 days, and tumor weight (B) was measured at endpoint. Antitumor immunity was assessed by single-cell RNA sequencing (C-Q). Genetic ablation of MrgD^+^ neurons reduced tumor growth (A) and tumor weight (B). C, Single-cell analysis of tumor-infiltrating leukocytes identified the major immune cell populations within the tumor. UMAPs are colored by annotated cell type. D,E, Cell-type composition of tumor-infiltrating leukocytes in control and MrgD-depleted tumors. F-P, Pathway and marker analyses of tumor-infiltrating immune cell states. Pseudobulk analyses show genotype-dependent changes across immune programs, including increased dendritic-cell activation after MrgD^+^ neuron ablation. Dot-plot analysis highlights increased expression of dendritic-cell activation markers, including Cd80, Cd86 and Cd40, in MrgD^+^ neuron-ablated tumors (P). Q, Gene-set enrichment analysis of significantly enriched pathways in control versus MrgD-depleted mice across whole immune, myeloid, T cell, NK-cell and B-cell compartments.

To determine whether distinct sensory neuron subsets differentially regulate tumor immunity, we next examined nonpeptidergic MrgD^+^ neurons, a molecularly and functionally distinct nociceptor population. We inducibly ablated MrgD^+^ neurons before orthotopic implantation of EO771-OVA tumors. Loss of MrgD^+^ neurons significantly reduced tumor growth and tumor weight, demonstrating that this sensory subset is sufficient to influence disease progression (Fig. 6A,B). Single-cell profiling of tumor-infiltrating leukocytes identified the major immune populations in both groups and showed that MrgD^+^ neuron ablation increased the proportion of cytotoxic NK cells while reducing the proportion of interferon-activated inflammatory macrophages (Fig. 6C-E).

Gene-set enrichment analysis of tumor-associated macrophages showed that pathways linked to TNFα/NF-κB signaling, JAK/STAT3 signaling, IFNα response, inflammatory responses, IL-2/STAT5 signaling and KRAS signaling were enriched in control tumors relative to MrgD-depleted tumors, indicating that MrgD^+^ sensory neurons sustain a distinct inflammatory macrophage program (Fig. 6Q). Consistently, differential gene-expression and pathway analyses revealed reduced *Il4ra* and Pycard expression alongside increased *Lyz2, Ifi44* and *Hspa1b* after MrgD^+^ neuron ablation, suggesting a shift away from IL-4-dependent polarization and toward interferon- and stress-associated activation states (Supplementary Fig. 7A-C). Pseudobulk analysis further showed increased dendritic-cell activation programs, including elevated *Cd80, Cd86* and *Cd40* expression, whereas selected M1-like antitumor signatures were reduced (Fig. 6F-P). Cell-cell communication analyses revealed broad remodeling of ligand-receptor interactions among immune populations after ablation of MrgD^+^ neurons, including macrophage-centered pathway changes and genotype-dependent differences in global communication networks (Supplementary Fig. 7D-F). Thus, MrgD^+^ neurons regulate a selective immune circuit characterized by altered macrophage signaling coupled to enhanced dendritic-cell activation.

### CGRP-RAMP1 signaling suppresses CD8^+^ T cell-mediated tumor control and limits response to immune checkpoint blockade

**Figure 7.**
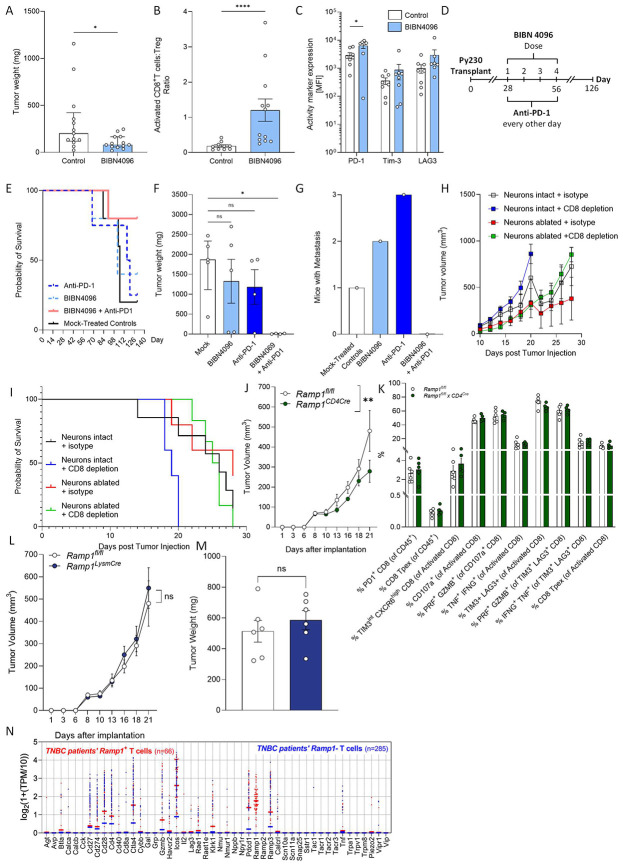
The CGRP-RAMP1 axis controls antitumor immunity A-C, Py230 cells (1 × 10^6^) were injected into the fourth mammary fat pad of 8-week-old female C57BL/6 mice. Beginning 28 days after tumor implantation, mice were randomized to receive the RAMP1 antagonist BIBN4096 (30 mg/kg, intratumoral, once weekly) or vehicle. Tumor growth (A) was monitored, and antitumor immunity (B,C) was assessed at endpoint by immunophenotyping of tumor-infiltrating leukocytes. RAMP1 antagonism reduced tumor growth (A), increased the ratio of activated CD8^+^ T cells to Treg cells (B), and increased PD-1 expression on activated CD8^+^ T cells (C), indicating that blockade of CGRP signaling through RAMP1 alters antitumor immunity. D-G, Py230 cells (1 × 10^6^) were injected into the fourth mammary fat pad of 8-week-old female C57BL/6 mice. Beginning 28 days after tumor implantation, mice were randomized to receive BIBN4096 (30 mg/kg, intratumoral, once weekly) or vehicle, with or without anti-PD-1 antibody (10 mg/kg, i.p., every other day). Treatment continued for 4 weeks after transplantation, and post-treatment survival was monitored for 84 days (D). Survival (E) was monitored for 130 days, and primary tumor weight (F) and lung metastatic burden (G) were assessed at endpoint by quantifying lung metastatic foci. Although BIBN4096 or anti-PD-1 monotherapy increased survival (E) and modestly reduced primary tumor growth (F), neither significantly affected metastatic burden (G). In contrast, combination treatment increased survival by approximately threefold and nearly abolished both primary tumor growth and lung metastasis, indicating that blockade of CGRP signaling through RAMP1 improves responsiveness to immune-checkpoint blockade. H,I, EO771-OVA cells (1 × 10^5^) were injected into the fourth mammary fat pad of 8-week-old female nociceptor-intact (Trpv1^wt/wt::DTA^fl/wt) or nociceptor-ablated (Trpv1-Cre^wt::DTA^fl/wt) mice. On day 3, mice in both groups were randomized to receive anti-CD8 antibody (200 μg per mouse, i.p., every 3 days; depletion assessed through day 14) or control treatment. Tumor growth (H) and survival (I) were monitored twice weekly for 28 days. n = 5 per group. J,K, EO771-OVA cells (2 × 10^5^) were injected into the fourth mammary fat pad of 8-week-old female mice with T cell-specific deletion of Ramp1 (Cd4-Cre^wt::Ramp1^fl/fl) or littermate controls (Cd4^wt/wt::Ramp1^fl/fl). Tumor growth (J) was monitored twice weekly for 18 days. Intratumoral CD8^+^ T cell phenotype was unchanged (K). Tumor growth was reduced in mice lacking Ramp1 in T cells, supporting a direct role for CGRP signaling in suppressing T cell-mediated antitumor immunity. L,M EO771-OVA cells (2 × 10^5^) were injected into the fourth mammary fat pad of 8-week-old female mice with Macrophage-specific deletion of Ramp1 (Ramp1^LysMCre^) or littermate controls (Ramp1^wt^). Tumor growth (L) was monitored twice weekly for 18 days and tumor weight (M) was assessed on day of sacrifice. Tumor growth remained unchanged in mice lacking Ramp1 in macrophages. N, In silico analysis of published single-cell RNA sequencing data from human TNBCs 41 showed that intratumoral RAMP1^+^ CD8^+^ T cells overexpress immune-checkpoint receptors, including PDCD1, HAVCR2 and CTLA4, as well as CD28, relative to RAMP1^−^ CD8^+^ T cells. These data indicate that the CGRP-RAMP1 axis also shapes antitumor immunity in human TNBC.

We next focused on the CGRP-expressing component of the tumor-associated sensory compartment and asked whether blocking CGRP signaling could phenocopy sensory denervation. In Py230 tumor-bearing mice, treatment with the RAMP1 antagonist BIBN4096 reduced tumor growth and increased the intratumoral ratio of activated CD8^+^ T cells to Treg cells (Fig. 7A,B). BIBN4096 also increased PD-1 expression on activated CD8^+^ T cells, indicating that CGRP signaling blockade reshapes the activation state and checkpoint profile of tumor-reactive T cells (Fig. 7C).

We then tested whether RAMP1 antagonism could improve responsiveness to immune-checkpoint blockade. BIBN4096 and anti-PD-1 monotherapy each increased survival and modestly reduced primary tumor growth, but neither treatment significantly reduced lung metastatic burden. In contrast, combined RAMP1 antagonism and PD-1 blockade produced a substantially stronger therapeutic effect, increasing survival by approximately threefold and nearly abolishing both primary tumor growth and lung metastasis (Fig. 7D-G). These data identify CGRP signaling as a therapeutically actionable pathway that limits response to immune-checkpoint blockade.

To directly test whether the antitumor effect of nociceptor ablation depends on CD8^+^ T cells, we depleted circulating CD8^+^ T cells in nociceptor-intact and nociceptor-ablated mice using neutralizing antibodies. Under control conditions, nociceptor ablation reduced tumor growth and improved survival. However, these protective effects were lost after CD8^+^ T cell depletion, demonstrating that sensory neurons promote tumor progression by limiting CD8^+^ T cell-dependent antitumor immunity (Fig. 7H,I). Flow cytometric analysis further showed that combined sensory neuron ablation and CD8^+^ T cell depletion reshaped the innate immune compartment, with reduced frequencies of ILC3s, total dendritic cells, Ly6C^+^ dendritic cells, and CD8^+^ dendritic cells, together with increased neutrophil frequency in specific neuron-ablated, CD8-depleted comparisons (Supplementary Fig. 8A-E). Consistent with a T cell-intrinsic role for CGRP sensing, T cell-specific deletion of *Ramp1* reduced tumor growth without detectably altering the measured intratumoral CD8^+^ T cell phenotype (Fig. 7J,K). In contrast, macrophage/myeloid-specific deletion of *Ramp1* using LysM-Cre did not affect tumor growth or tumor volume, supporting a primarily T cell-driven mechanism (Fig. 7L,M).

Finally, analysis of published human TNBC single-cell RNA-seq data showed that intratumoral RAMP1^+^ CD8^+^ T cells expressed higher levels of PDCD1 (encoding PD-1), HAVCR2 (encoding TIM-3), CTLA4 and CD28 than RAMP1^−^ CD8^+^ T cells (Fig. 7L). Together, these findings establish a nociceptor-CGRP-RAMP1 signaling axis that suppresses antitumor T cell function and can be targeted to enhance immunotherapy efficacy.

## DISCUSSION

These findings establish tumor-innervating nociceptors as active regulators of breast cancer immunity. Previous studies have shown that nerves are not incidental bystanders in mammary tumors: PGP9.5^+^ neural invasion in breast carcinoma has been associated with aggressive disease and shorter disease-free survival, intratumoral nerve fibers have been linked to lymph node invasion and tumor-derived NGF, and breast cancer-conditioned medium can drive sensory neuritogenesis and CGRP-associated nociceptor sprouting^32,48,49^. We extend this framework by showing that TNBC lesions and their draining lymph nodes are densely innervated by CGRP^+^ nociceptors, and by identifying a downstream immune consequence of this innervation: a tumor-derived proNGF–NGFR axis that amplifies CGRP release and suppresses CD8^+^ T cell cytotoxicity. These data complement independent evidence that nociceptor neuron, in this case via a substance P–TACR1–extracellular ssRNA–TLR7 axis, regulates breast cancer progression and metastasis^50^.

More broadly, our findings position TNBC within a growing class of CGRP-sensitive neuroimmune malignancies, alongside melanoma^17^, gastric cancer^36^, head and neck squamous cell carcinoma^18^, and lung adenocarcinoma^35^. In melanoma, tumor cells promote nociceptor neurite outgrowth, neuronal activation, and neuropeptide release, while nociceptors drive tumor progression by promoting CD8^+^ T cell exhaustion through CGRP–RAMP1 signaling. In lung adenocarcinoma, tumor progression similarly amplifies local nociceptive innervation and CGRP release, which acts on macrophages to suppress CXCL13^+^ fibroblast recruitment, tertiary lymphoid structure formation, and B cell- and T cell-dependent antitumor immunity. Sensory denervation or pharmacologic CGRP blockade restores TLS formation, restrains tumor growth, and sensitizes cigarette smoke-exposed tumors to immunotherapy, revealing a neuroimmune mechanism by which smoking accelerates LUAD progression independently of somatic mutagenesis^35^. In head and neck cancer, sensory nerve-derived CGRP also promotes tumor growth by suppressing tumor-infiltrating lymphocytes. Our breast cancer data extend this framework by showing that pharmacologic RAMP1 blockade, T cell-specific Ramp1 deletion, and nociceptor ablation each relieve immune suppression and improve tumor control. Moreover, the synergy with anti-PD-1 suggests that CGRP functions as a neural checkpoint layered onto canonical immune checkpoints. Thus, nociceptor activity is not merely a correlate of cancer pain, but an active determinant of immune dysfunction in the tumor microenvironment.

At the same time, CGRP is not intrinsically immunosuppressive; outside cancer, it functions as a context-dependent neuroimmune rheostat. In bacterial pneumonia, TRPV1^+^ nociceptors suppress protective immunity and bacterial clearance by limiting neutrophil and γδ T cell responses^51^. In Staphylococcus aureus skin infection, TRPV1^+^ neurons inhibit neutrophil recruitment and regulate macrophage polarization through CGRP^52^ . By contrast, optogenetic activation of cutaneous TRPV1^+^ neurons can elicit protective type 17 immunity that enhances host defense against Candida albicans and S. aureus^53^, and in sterile tissue injury, CGRP-RAMP1 signaling in neutrophils, monocytes and macrophages promotes resolution and wound repair^54,55^. Our findings place breast cancer on the chronic-hijacking side of CGRP biology, in which a normally homeostatic and tissue-protective mediator is repurposed to facilitate immune escape.

The dense sensory innervation observed in tumor-draining lymph nodes raises the possibility that CGRP shapes not only intratumoral effector function but also earlier steps in T cell priming, maintenance or trafficking. Although our CD8-depletion and T cell-specific Ramp1 experiments establish a direct lymphoid arm, the broad remodeling of dendritic cells and macrophages after denervation suggests that breast tumors engage a multicellular CGRP network. The coexistence of CGRP-driven immune suppression and substance P-driven pro-metastatic signaling further suggests that sensory neurons, possibly overlapping peptidergic subset, may coordinate separable but cooperative programs: one that disables cytotoxic immunity and another that enhances metastatic competence. Therapeutically, this argues for combining checkpoint blockade with selective interruption of tumor-activated neuropeptide circuits, rather than viewing cancer-associated innervation as only a symptomatic feature of pain. More broadly, our data identify tumor-innervating nociceptors as actionable components of the breast tumor microenvironment and support neuroimmune signaling as a therapeutic axis in TNBC.

## METHODS

### Mouse lines and husbandry

All experimental procedures were approved by the OUHSC Institutional Animal Care and Use Committee and performed in accordance with US National Institutes of Health guidelines. Female mice (~1-6 months of age; ~20-28 g) were group housed under a 12 h:12 h light:dark cycle (lights on at 7:00 a.m.) in a room maintained at ~22 °C and ~45% humidity. Food (LabDiet, #LD505330LB) and water were provided ad libitum. All mouse lines were maintained on a C57BL/6J background unless otherwise indicated. Breeding pairs were originally purchased from The Jackson Laboratory and included C57BL/6J (wild-type, #000664), B6.129-Scn10^tm2(cre)Jwo/TjpJ (Nav1.8-Cre, #017293), B6N;129-Tg(CAG-CHRM3*,-mCitrine)1Ute/J (LSL-hM3Dq, #026220), C3(1)-TAg (#013591), B6.Cg-Gt(ROSA)26Sor^tm14(CAG-tdTomato)Hze/J (LSL-tdTomato, #007914), B6.129-Trpv1^tm1(cre)Bbm/J (TRPV1-Cre, #017769), Mrgprd^tm1.1(cre/ERT2)Wql/J (MrgD-Cre, #031286), B6.129P2-Gt(ROSA)26Sor^tm1(DTA)Lky/J (LSL-DTA, #009669) and BALB/cJ (#000651).

### Cancer cell handling and orthotopic transplantation

The Py230 triple-negative breast cancer cell line was obtained from the American Type Culture Collection and cultured in T-185 flasks according to the supplier's recommendations. On the day of transplantation, cells were harvested, and viable cells were counted using ViaStain AOPI Staining Solution (Nexcelom Bioscience, CS2-0106) and a CellDrop automated cell counter (DeNovix). Cells were resuspended 1:1 in Matrigel matrix (Corning, 354262), and 1 × 10^6^ cells were injected into the right fourth mammary fat pad.

EO771 cells were purchased from ATCC (The Global Bioresource Center; #CRL-3461) and cultured in Dulbecco's modified Eagle's medium (DMEM) supplemented with 10% fetal bovine serum (FBS) and 1% penicillin-streptomycin. Cells were incubated at 37 °C in 5% CO_2_ and 95% air. Before injection, EO771 cells (~80-90% confluence) were detached with trypsin, and cell number and viability were determined with a 1:1 mixture of trypan blue (Life Technologies) using an automated hemocytometer (Countess II, Life Technologies, Carlsbad, CA, USA). Cells were diluted in PBS and mixed 1:1 with growth factor-reduced Matrigel Matrix (BD Biosciences, Bedford, MA, USA) on ice to a final concentration of 2.5 × 10^5^ cells per 100 μL before administration.

4T1.2 cells were cultured in 10-cm tissue-culture-treated dishes (USA Scientific) at 37 °C with 5% CO_2_ in DMEM (Gibco) supplemented with 10% fetal bovine serum (Life Technologies) and 1% penicillin-streptomycin (Fisher Scientific). Cells were passaged using 0.25% trypsin (Corning) and used at passage numbers below 16. Before injection, cells were detached, counted and diluted to 1 × 10^5^ cells per 30 μL medium without Matrigel. Primary epidermal keratinocytes (PEK; Cell Biologics), used as a non-malignant comparator, were cultured in Complete Epithelial Cell Medium Kit medium containing base medium, 0.5 mL epidermal growth factor, 0.5 mL hydrocortisone, 1% antibiotic-antimycotic and 2% FBS (Cell Biologics). PEK cells were passaged and maintained according to the vendor's instructions using gelatin-based coating and Trypsin-EDTA 0.05% Solution (Cell Biologics).

4T1 breast tumor cells (kindly provided by Dr. Audrey Klange's laboratory at the University of Montreal) were maintained in RPMI-1640 medium (Corning, 10-040-CV) supplemented with 10% FBS (Seradigm, 3100) and 1% penicillin-streptomycin (Corning, MT-3001-Cl). Cells were cultured at 37 °C in a humidified incubator with 5% CO_2_. On the day of transplantation, cancer cells were resuspended in phosphate-buffered saline (PBS; Corning, 21-040-CV) and injected into the fourth mammary fat pad (5 × 10^5^ cells in 100 μL).

Mice were briefly anesthetized with isoflurane and received injections of EO771 cells (C57BL/6J mice) or 4T1/4T1.2 cells (BALB/c mice) into the right inguinal mammary fat pad or vehicle alone. Proper injection was confirmed by the formation of a visible wheal. Mice were monitored approximately every 2-3 days for tumor growth. Once tumors became palpable, measurements were taken daily using sliding calipers by the same investigator, and tumor volume was calculated using the formula: volume = (length × width^2^)/2. Mice were excluded from the study and euthanized if tumor ulceration occurred. At endpoint, tumors were fixed in 4% paraformaldehyde (PFA) for 48 h at 4 °C, washed with PBS and processed for downstream analyses.

### Immunohistochemical characterization of mammary fat pad-innervating sensory afferents

One week before inoculation, BALB/c mice were briefly anesthetized with 2% isoflurane (Covetrus), and 20 μL of 2% Fast Blue (FB; Polysciences) was injected subcutaneously into the fourth mammary fat pad to retrogradely label innervating neurons. On post-inoculation days 24-27, T10-T13 DRGs were isolated from 4T1.2-bearing or sham mice. Mice were anesthetized with isoflurane, perfused with cold PBS, and ipsilateral DRGs were dissected. DRGs were post-fixed in 4% paraformaldehyde (PFA; Electron Microscopy Sciences) for 24 h, cryoprotected in 30% sucrose, embedded in OCT compound (Tissue-Tek) and cryosectioned at 10 μm onto Superfrost Plus slides (Thermo Fisher Scientific). Sections were blocked for 1 h at room temperature (RT) in 10% normal goat serum (Gibco), 0.01% Triton X-100 (Sigma-Aldrich), 1% bovine serum albumin (Fisher Scientific) and 0.1% Tween-20 (Sigma-Aldrich). Tissue was incubated in primary antibody diluted in blocking buffer for 3 days at 4 °C [rat anti-substance P (1:200; BD Pharmingen), rabbit anti-TRPV1 (1:1000; Alomone)] or overnight [IB4-Alexa Fluor 594 (1:1000; Sigma-Aldrich), rabbit anti-CGRP (1:250; Cell Signaling)], followed by secondary antibody diluted 1:250 in blocking buffer for 2 h at RT [goat anti-rabbit Alexa Fluor 594 (Jackson ImmunoResearch), anti-rat 800 (Invitrogen)]. Slides were coverslipped with Fluoro-Gel mounting medium (Electron Microscopy Sciences), and images were acquired at 20× magnification on a Keyence BZ-X810 microscope. Positively stained ganglion neurons with distinct nuclei and at least 50% of the cell area labeled with FB were manually counted from 10 sections, 100 μm apart, for each DRG. Images were counted by a blinded reviewer.

### Calcium imaging of mammary fat pad-innervating sensory afferents

DRG dissociation: Three weeks before calcium imaging, BALB/c mice were briefly anesthetized with isoflurane, and 20 μL of the retrograde tracer 1,1'-dioctadecyl-3,3,3',3'-tetramethylindocarbocyanine perchlorate (DiI, Invitrogen) was injected into the fourth mammary fat pad. DiI was dissolved at 170 mg/mL in DMSO (Thermo Fisher Scientific) and diluted 1:10 in 0.9% sterile saline (Hospira). Mice were perfused with PBS, and DRGs were dissected and dissociated for Ca^2+^ imaging as previously described^56^. Briefly, tissue was harvested into HBSS+H and subjected to two-step enzymatic digestion with L-cysteine and papain (Sigma-Aldrich) for 10 min at 37 °C, followed by combined collagenase type II and dispase II (Gibco) digestion for 20 min at 37 °C. Tissue was centrifuged at 240 × g, resuspended in DMEM/F12 (Gibco), passed through a 40 μm filter and plated on 12-mm glass coverslips (Electron Microscopy Sciences) coated with poly-L-ornithine and laminin (Sigma-Aldrich).

Conditioned media collection: 4T1.2 or PEK cells were cultured in 10-cm dishes (see above) to 80-90% confluence. Media were removed and replaced with 3 mL of serum-free, phenol red-free medium for 48 h [DMEM without phenol red (Gibco) supplemented with 1% penicillin-streptomycin]. Conditioned media were collected, centrifuged at 400 × g to remove dead cells and debris, and the supernatant was applied during calcium imaging.

Calcium imaging: Acutely dissociated DRG neurons were incubated with 5 μM Fura-2AM (Fura, Invitrogen), a Ca^2+^ indicator, for 30 min at RT. Coverslips were washed for 5 min in normal bath solution (NB; 130 mM NaCl, 3 mM KCl, 2.5 mM CaCl_2_, 0.6 mM MgCl_2_, 10 mM HEPES, 10 mM glucose, pH 7.4, 325 mOsm). Coverslips were then placed in the recording chamber and continuously perfused with clean culture medium. Baseline Ca^2+^ activity was measured for 5 min before stimulation. 4T1.2 or PEK conditioned medium was applied for 60 s, followed by KCl (30 mM; 4 s) to determine viability and capsaicin (500 nM; 4 s). Cells were allowed to re-establish baseline between applications. Response to stimulus was defined as ΔF ≥ 20% of baseline. Response magnitude was calculated as peak ΔF minus baseline ΔF. Fluorescence data were acquired on a Leica DMi8 microscope at 340 nm and 380 nm excitation wavelengths and analyzed with Leica Application Suite software (Leica Microsystems).

### Primary mouse dorsal root ganglion (DRG) neuron isolation and culture

Primary dorsal root ganglion (DRG) neurons were isolated from postnatal day 3 to day 10 (P3-P10) C57BL/6J mice using an established protocol^57^. Mice were euthanized according to institutional animal care and use guidelines, and spinal columns were rapidly dissected and transferred into ice-cold Ca^2+^/Mg^2+^-free Hank's Balanced Salt Solution (HBSS). DRGs were collected bilaterally under a stereomicroscope, with careful removal of meninges, nerve roots and connective tissue.

Isolated ganglia were enzymatically dissociated first with collagenase IV (2 mg/mL; 1:20 dilution in DMEM/F-12) for 30 min at 37 °C, followed by 2.5% trypsin for 30 min at 37 °C. Enzymatic reactions were terminated by washing ganglion pellets twice with pre-warmed BrainPhys neuronal culture medium (STEMCELL Technologies, Cat# 05790)^58,59^. The partially dissociated tissue was then mechanically triturated using fire-polished glass pipettes of decreasing bore size to obtain a single-cell suspension. The cell pellet was resuspended in BrainPhys medium supplemented with 20 ng/mL NGF^60^ and penicillin-streptomycin. Cells were plated onto poly-D-lysine (PDL)- and laminin-coated 24-well plates at densities optimized for downstream applications. Cultures were maintained at 37 °C in a humidified incubator with 5% CO_2_ in BrainPhys medium, with half-medium changes every 2-3 days using pre-warmed medium. Neurons were allowed to stabilize for 72 h before experimental treatments.

### BIBN4096 monotherapy

For BIBN4096 monotherapy experiments, C57BL/6 mice were orthotopically implanted with Py230 cells in the fourth mammary fat pad. Beginning 28 days after tumor implantation, mice received intratumoral injections of BIBN4096 (30 mg/kg; MedChemExpress, HY-10095) or vehicle control (DMSO) once every 7 days for 4 weeks. Mice were euthanized 60 days after transplantation, and tumors, lymph nodes and spleens were collected for flow cytometric analysis.

### BIBN4096 and immune-checkpoint blockade combination treatment

For combination-treatment experiments, C57BL/6 mice were orthotopically implanted with Py230 cells in the fourth mammary fat pad. Beginning 28 days after tumor implantation, mice received intratumoral injections of BIBN4096 (30 mg/kg; MedChemExpress, HY-10095) or DMSO once every 7 days for 4 weeks. In parallel, mice were treated intraperitoneally every other day for 4 weeks with anti-PD-1 antibody (10 mg/kg; Bio X Cell, RMP1-14) or IgG isotype control antibody (10 mg/kg; Bio X Cell, 2A3). Mice were monitored daily for survival and morbidity. In accordance with OUHSC IACUC guidelines, animals were euthanized when tumors reached a maximum diameter of 15 mm in any dimension. At endpoint, tumors were harvested, weighed and fixed in 10% formalin.

### DREADD-mediated sensory neuron activation

For DREADD experiments, 1 × 10^6^ EO771 cells were orthotopically injected into the right fourth mammary fat pad to induce tumor formation. Tumor monitoring was performed daily beginning on day 10 after tumor inoculation. The longer and shorter tumor dimensions were measured and used to calculate tumor volume. Body weight, food intake and water intake were also monitored daily.

To induce sensory neuron activation, mice received daily intraperitoneal injections of clozapine-N-oxide (CNO; 1 mg/kg) or deschloroclozapine (DCZ; 3 μg/kg) until euthanasia on day 28 after tumor inoculation. Randall-Selitto and tail-flick assays were performed on days 9 and 27 after tumor inoculation to assess sensory neuron activation.

### Capsaicin- and resiniferatoxin-mediated sensory neuron ablation

Chemical ablation of TRPV1^+^ sensory neurons was performed using capsaicin or resiniferatoxin (RTX). For capsaicin-mediated ablation, 8-week-old mice received intraperitoneal injections of capsaicin once daily for 3 consecutive days. Capsaicin was prepared in saline and administered at 100 μM in 100 μL per mouse. EO771 breast cancer cells were orthotopically implanted into the mammary fat pad 3 days after the final capsaicin injection. For implantation, 2.5 × 10^5^ EO771 cells were resuspended in a 1:1 mixture of saline and Matrigel. Body weight and tumor volume were measured every 3 days for 27 days. For RTX-mediated ablation, 4-week-old mice received intraperitoneal injections of RTX at escalating doses of 30, 70 and 100 μg/kg over 3 consecutive days. RTX was prepared in saline. Four weeks after RTX treatment, 2.5 × 10^5^ EO771 cells, resuspended in a 1:1 mixture of saline and Matrigel, were orthotopically implanted into the mammary fat pad. Tumor volume was measured every other day for 27 days.

### Assessment of sensory nerve depletion and overactivation

To validate sensory nerve depletion and overactivation, behavioral nociceptive assessments were performed at baseline, before orthotopic EO771 breast cancer cell inoculation, and on post-inoculation days 9 and 27. To minimize experimental stress and confounding behavioral variability, all mice were habituated to the testing environment and apparatus daily for 3 consecutive days before data collection. For mechanical and thermal modalities, the definitive nociceptive threshold was calculated as the mean of 3 consecutive trials per animal.

### Mechanical nociception (Randall-Selitto test)

Mechanical withdrawal thresholds were evaluated using the Randall-Selitto paw-pressure test^61^. Stimuli were delivered with an algesimeter equipped with a dome-shaped, blunt-tipped cone plunger (Ugo Basile, Italy) applied to the dorsal surface of the hind paw. A linearly increasing mechanical force was applied, and the mechanical threshold, defined as the pressure (g) and latency (s) required to elicit a clear nociceptive reflex (for example, paw withdrawal or vocalization), was recorded. A strict cutoff force of 160 g was used in all trials to prevent mechanical tissue damage.

### Thermal nociception (tail-flick test)

Thermal nociceptive thresholds were determined using a radiant-heat tail-flick assay^62^. The distal 2 cm of the tail was positioned over a focused radiant-heat source. Tail-flick latency, defined as the duration from onset of thermal stimulation to a sharp, reflexive tail withdrawal, was recorded automatically. A maximum cutoff latency of 8 s was imposed to avoid thermal tissue injury.

### ProNGF neutralization in an orthotopic breast tumor model

Eight-week-old C57BL/6 mice bearing orthotopic EO771 tumors were randomized to receive control antibody or proNGF-neutralizing monoclonal antibody (pNEUT). This monoclonal antibody inhibits proNGF binding and activation of p75NTR. Antibodies were administered intraperitoneally at 75 μg per dose on days 7, 14 and 21 after tumor implantation. Each group included eight mice^63^.

### RNA extraction and quantitative RT-PCR

Total RNA was extracted using TRIzol reagent (Invitrogen) according to the manufacturer's protocol, including chloroform phase separation, isopropanol precipitation and 75% ethanol washes. RNA quality and quantity were assessed by spectrophotometry using a NanoDrop instrument. Complementary DNA was synthesized using MMLV reverse transcriptase (Lucigen) according to standard reaction conditions.

Quantitative PCR was performed using TaqMan Fast Advanced Master Mix (Life Technologies), containing AmpliTaq Fast DNA Polymerase, in 10 μL triplex reactions with TaqMan gene-specific probes. Each reaction used 50 ng cDNA. Amplification was performed under standard cycling conditions. Relative gene expression was calculated using a standard curve generated from serially diluted pooled cDNA samples and normalized to 18S rRNA. All reactions were performed in technical replicates, and no-template controls were included to exclude contamination.

### Bulk RNA sequencing and data analysis

Total RNA was extracted from monocultures of EO771 cancer cells or primary dorsal root ganglion neurons, as well as from FACS-isolated cancer cells and neurons, using TRIzol reagent. RNA was purified by isopropanol precipitation and ethanol washing. RNA quality and quantity were assessed using a TapeStation system (Agilent).

cDNA libraries were prepared using the Kapa mRNA HyperPrep Kit for Illumina Platforms (Roche, 08098115702) according to the manufacturer's protocol. Sequencing was performed using the NextSeq 2000 P1 300-cycle kit (Illumina), with a target output of 200 million paired-end reads.

Bulk RNA-seq data were analyzed using a JupyterHub-based pipeline. Initial quality control of raw sequencing data was performed using FastQC. Reads were aligned to the mouse reference genome GRCm39 using STAR, and gene-level quantification was performed using featureCounts. Differential expression analysis was conducted using DESeq2. Genes with a false discovery rate below 0.05 were considered differentially expressed.

Raw sequencing data and processed expression matrices have been deposited in the NCBI Gene Expression Omnibus under accession number GSE330238.

### CGRP quantification by ELISA

Calcitonin gene-related peptide levels were quantified in orthotopic breast tumor tissues using a competitive CGRP ELISA kit (Bertin Bioreagent, A05482-CGRP [rat]) according to the manufacturer's instructions.

Briefly, tumor tissues were harvested, weighed and homogenized in 2 N acetic acid supplemented with protease inhibitors. Homogenates were centrifuged, and supernatants were collected for analysis. Samples and standards were added to antibody-coated microplate wells, followed by enzyme tracer. After washing, substrate was added, and plates were incubated for 1.5 h at room temperature on an orbital shaker in the dark. Absorbance was measured at 414 nm using a microplate reader. CGRP concentrations were calculated from standard curves and normalized to tumor tissue weight. All samples were assayed in technical duplicates, and analyses were performed blinded to experimental group allocation.

### BABB tissue clearing

Three-dimensional visualization of tumor innervation was performed using benzyl alcohol/benzyl benzoate tissue clearing. Briefly, tissues were fixed in paraformaldehyde, washed in PBS and permeabilized. Samples were then incubated with primary and secondary antibodies under gentle agitation at 4 °C, followed by extensive washing in PBS. After immunostaining, tissues were dehydrated through a graded methanol series and incubated sequentially in a 1:1 mixture of methanol and dichloromethane, followed by dichloromethane alone for lipid removal. Samples were then transferred to BABB solution, consisting of benzyl alcohol and benzyl benzoate at a 1:2 volume ratio, for refractive-index matching and optical clearing. Cleared tissues were imaged by confocal microscopy. Unless otherwise specified, all steps were performed at room temperature, and samples were protected from light throughout the procedure.

### Single-cell RNA sequencing

EO771-OVA tumors harvested from MrgD-CreER and MrgD-wild-type mice were used to prepare single-cell suspensions. Tumors were mechanically and enzymatically dissociated, and immune cells were enriched using a Percoll density gradient. Individual samples corresponded to separate mice and included eight MrgD-CreER and six MrgD-wild-type biological replicates.

Single-cell RNA-seq libraries were generated using the Chromium Next GEM Single Cell Fixed RNA Sample Preparation Kit (10x Genomics, PN-1000414) according to the manufacturer's instructions. Briefly, fixed single-cell suspensions were processed on the Chromium platform to generate Gel Bead-in-Emulsions, enabling barcoding of individual cells and transcripts. Libraries were then constructed and sequenced on a high-throughput Illumina platform. Raw sequencing data were processed to generate gene-expression count matrices for downstream single-cell analysis.

Single-cell sequencing was performed on tumor and lymph node samples from TRPV1-Cre (neuron-intact) and TRPV1-Cre × DTA (neuron-ablated) mice. Inguinal lymph node samples were pooled from four mice per group, digested with the Miltenyi Spleen Dissociation Kit, and sorted as CD45^+^7AAD^−^ cells. Tumor samples were digested with the Miltenyi Tumor Dissociation Kit and sorted as CD45^+^7AAD^−^ cells. Tumor samples were then fixed, barcoded and pooled, allowing analysis of pooled samples or individual samples. Single-cell suspensions were loaded onto the 10x Genomics Chromium X for capture in droplet emulsion. Libraries were prepared using the Chromium GEM-X Single Cell 3' Kit v4 according to the standard protocol.

Libraries were sequenced on an Illumina NextSeq 2000 according to 10x Genomics recommendations. 10x Genomics Cell Ranger 6.0 was used for demultiplexing, alignment, counting, clustering and differential expression. Prepared data were viewed and exported using 10x Genomics Loupe Browser. [AUTHOR QUERY: complete this sentence if additional export or analysis steps were used.]

### Single-cell RNA-seq analysis

Single-cell RNA-seq data from two independent tumor models were analyzed using Scanpy. The MrgD dataset included cells from EO771-OVA breast tumors from MrgD-CreER and MrgD-wild-type mice. The TRPV1-DTA dataset included cells from EO771 breast tumors from control (TRPV1-Cre) and TRPV1-Cre × DTA tumor-bearing mice, with four biological replicates per group (1 × 10^5^ EO771 cells were injected).

Raw count matrices from individual samples were loaded and merged into a single object for each dataset. Quality-control filtering was performed to remove low-quality cells. Cells were retained if they had 1,000-10,000 detected genes, less than 20% mitochondrial gene content, less than 1% hemoglobin gene expression and fewer than 100,000 total counts.

Cells were clustered using the Leiden algorithm, and marker genes were identified for downstream annotation. Cell-type annotation was performed using the automated CyteType workflow, which assigns cell identities based on top marker genes per cluster. Automated annotations were subsequently verified using canonical marker genes and manually curated into major immune cell populations. UMAP embeddings were used to visualize cellular heterogeneity.

Cell-type composition was calculated as the proportion of each annotated cell type normalized to the total number of cells within each condition.

To assess immune functional states, gene module scores were computed using curated gene sets representing key immunological programs. These included dendritic-cell activation, dendritic-cell migration, T cell response, T cell effector function, T cell exhaustion, immune tolerance, M1-like macrophage polarization and M2-like macrophage polarization.

The following gene sets were used:

**Table:** 

Module	Genes
Dendritic cell activation	Cd80, Cd86, Cd40
Dendritic cell migration	Ccr7, Cxcl9, Cxcl10, Ccl7
T cell response	Cd28, Icos, Tnfrsf9
T cell effector function	Gzmb, Prf1, Ifng, Tnf, Fasl
T cell exhaustion	Pdcd1, Tox, Lag3, Havcr2, Eomes
Immune tolerance	Ctla4, Tigit, Ido1, Entpd1, Foxp3
M1-like macrophages / anti-tumor macrophage program	Cxcl9, Cxcl10, Nos2, Il12b, Cd86, Tnf, Ccl5
M2-like macrophages / pro-tumor macrophage program	Arg1, Mrc1, Il10, Tgfb1, Cd163, Retnla

Only genes present in the dataset were included in module scoring. Module scores were visualized on UMAP embeddings and compared between conditions at both the single-cell and pseudobulk levels. Single-cell comparisons were performed using the Mann-Whitney U test. For pseudobulk analysis, module scores were averaged per sample and compared using Welch's t-test. Dot plots were used to summarize gene-expression patterns across conditions. Dot size represents the fraction of cells expressing a given gene, and color intensity indicates scaled mean expression. For these single-cell module analyses, statistical testing was performed without multiple-testing correction.

## Tissue processing and morphometric analysis

Skeletal muscles from sensory neuron-denervated animals were harvested, dissected across the mid-belly region and fixed in 4% buffered paraformaldehyde (PFA, pH 7.4) for 4 h. Following fixation, tissues were transferred to 70% ethanol, embedded in paraffin and sectioned transversely at 5 μm. Sections were stained with hematoxylin and eosin (H&E) according to standard protocols. Representative brightfield images were acquired at 20× magnification using an Olympus BX50 light microscope equipped with an Olympus Q-Color3 digital camera system. Morphometric evaluation, including myofiber cross-sectional area^64^, minimum Feret diameter, and the presence of centralized or misplaced nuclei, was performed using Fiji/ImageJ software (version 1.54; National Institutes of Health, USA)^65^. A minimum of 200 myofibers across five or more randomly selected fields were analyzed per animal sample. To ensure consistent spatial classification, nuclei were categorized based on centroid position relative to the muscle fiber boundary. Any nucleus located internally relative to the sarcolemma boundary, whether proximal to the border or within the fiber core, was defined as a central nucleus. The proportion of internally nucleated muscle fibers was calculated and expressed as a percentage of the total number of myofibers analyzed per sample^66,67^.

### Antibody-mediated depletion and checkpoint-inhibitor blockade

Mice were treated intraperitoneally with 300 μg anti-CD8 antibody (Bio X Cell) or the recommended isotype control on days -1, 0, 3 and 7 after tumor injection and then once weekly after day 7. For checkpoint-inhibitor blockade, mice were treated intraperitoneally with 250 μg anti-PD-1 antibody (Bio X Cell) or the recommended isotype control on days 10, 12 and 14 after tumor injection and then every 4 days.

### Spheroid generation and co-culture with DRGs

Tumors were collected from MMTV-PyMT mice once they reached a width or length of 10 mm. C57BL/6J mice were used to generate control mammary gland spheroids. Following a published protocol^68^, tumor and control spheroids were generated and plated at 50 spheroids per 50 μL Matrigel dome. For co-culture experiments, DRGs were isolated from control mice and added directly to the Matrigel (Corning) dome. Once the Matrigel solidified, medium was added to cover the dome as described^68^. Medium was changed once per week.

### Neuronal tract tracing

Neuronal tract tracing was performed using [AUTHOR QUERY: add tracer, injection site, dose or concentration, survival time, tissue-processing method, imaging approach and analysis method].

### Flow cytometry

To analyze tumor-infiltrating immune cells, triple-negative breast tumors from C57BL/6 mice were harvested, minced and transferred to digestion buffer containing collagenase IV (Gibco, 17104-019; 1.5 mg/mL) and DNase I (Thermo Scientific, EN0523; 1 U/μL). Tumors were digested for 45 min at 37 °C with gentle agitation. After digestion, tissues were washed and resuspended in RPMI 1640 medium to generate single-cell suspensions.

Cells were stained for 1 h at 4 °C in the dark with fluorescently conjugated antibodies listed in Table 1. Intracellular staining was performed using the Cytofix/Cytoperm fixation/permeabilization kit (BD Biosciences, 51-2090KZ) according to the manufacturer's instructions. Cells were stained intracellularly with FoxP3 for 1 h at 4 °C, washed twice with permeabilization buffer, washed once with PBS and fixed in 2% paraformaldehyde.

### Statistical analysis

Data are presented as mean ± SEM unless otherwise indicated. Statistical analyses were performed using GraphPad Prism version 10.4.1. Datasets were first assessed for normality and outliers. Comparisons between two groups were performed using a two-tailed unpaired t-test or a Mann-Whitney U test, depending on data distribution. Comparisons among more than two groups were performed using one-way or two-way ANOVA for normally distributed datasets, followed by appropriate post hoc tests. Nonparametric datasets were analyzed using the Kruskal-Wallis test followed by Dunn's post hoc test for multiple comparisons. A p value of ≤ 0.05 was considered statistically significant. Where appropriate, p values were adjusted for multiple comparisons.

## Supplementary Material

Supplement 1

## Figures and Tables

**Table 1.** Markers used for flow cytometric staining

Antigen	Fluorophore	Concentration	Clone	Vendor
Zombie Aqua	BV510	1:100	77143	BioLegend
CD45	FITC	1:150	30-F11	BioLegend
CD44	PE	1:150	IM7	BioLegend
CD25	PE-Cy5	1:200	PC61	BioLegend
CD43	PE-Cy7	1:150	1B11	BioLegend
CD8	BV605	1:200	53-6.7	BioLegend
CD4	BV650	1:200	RM4-5	BioLegend
TIM-3 / CD366	APC	1:150	B8.2C12	BioLegend
PD-1	PE-Dazzle594	1:150	RMP1-30	BioLegend
LAG-3 / CD223	PerCP-Cy5.5	1:150	C9B7W	BioLegend
IgM	PerCP-Cy5.5	1:500	RMM-1	BioLegend
CD86	BV785	1:150	GL-1	BioLegend
FAS / CD95	BV605	1:200	SA367H8	BioLegend
MHC class II	BV650	1:2000	M5/114.15.2	BioLegend
CXCR4 / CD184	BV711	1:150	L276F12	BioLegend
B220	APC-Fire750	1:150	RA3-6B2	BioLegend
CD80	PE-Dazzle594	1:150	16-10A1	BioLegend
GL-7	PE-Cy7	1:150	GL7	BioLegend
IgD	APC	1:150	11-26c.2a	BioLegend
B220	AF-488	1:200	RA3-6B2	BioLegend
CD3	PE	1:150	17A1	BioLegend
CD24	PE-Cy5	1:1500	M1/69	BioLegend
F4/80	PE-Cy7	1:200	BM8	BioLegend
CD64	BV421	1:200	X54-5/7.1	BioLegend
CD45	BV605	1:200	30-F11	BioLegend
Ly6C	BV711	1:200	HK1.4	BioLegend
CD11c	BV785	1:200	N418	BioLegend
Ly6G	AF700	1:200	1A8	BioLegend
CD11b	APC-Fire750	1:200	M1/70	BioLegend
CD4	PE-Dazzle594	1:200	RM4-5	BioLegend
FoxP3	BV421	1:200	MF-14	BioLegend
