# Supplementary material for: Nociceptor neurons suppress antitumor immunity in breast cancer
Source: Res Sq. 2026 Aug 17:rs.3.rs-9927184. Originally published 2026 Jun 9. Preprint. [Version 2] doi: 10.21203/rs.3.rs-9927184/v2 (PMC13278325; doi:10.21203/rs.3.rs-9927184/v2)
Supplement: Supplementary file 1 [file NIHPPrs9927184v2-supplement-1.pdf]

Supplementary Figures

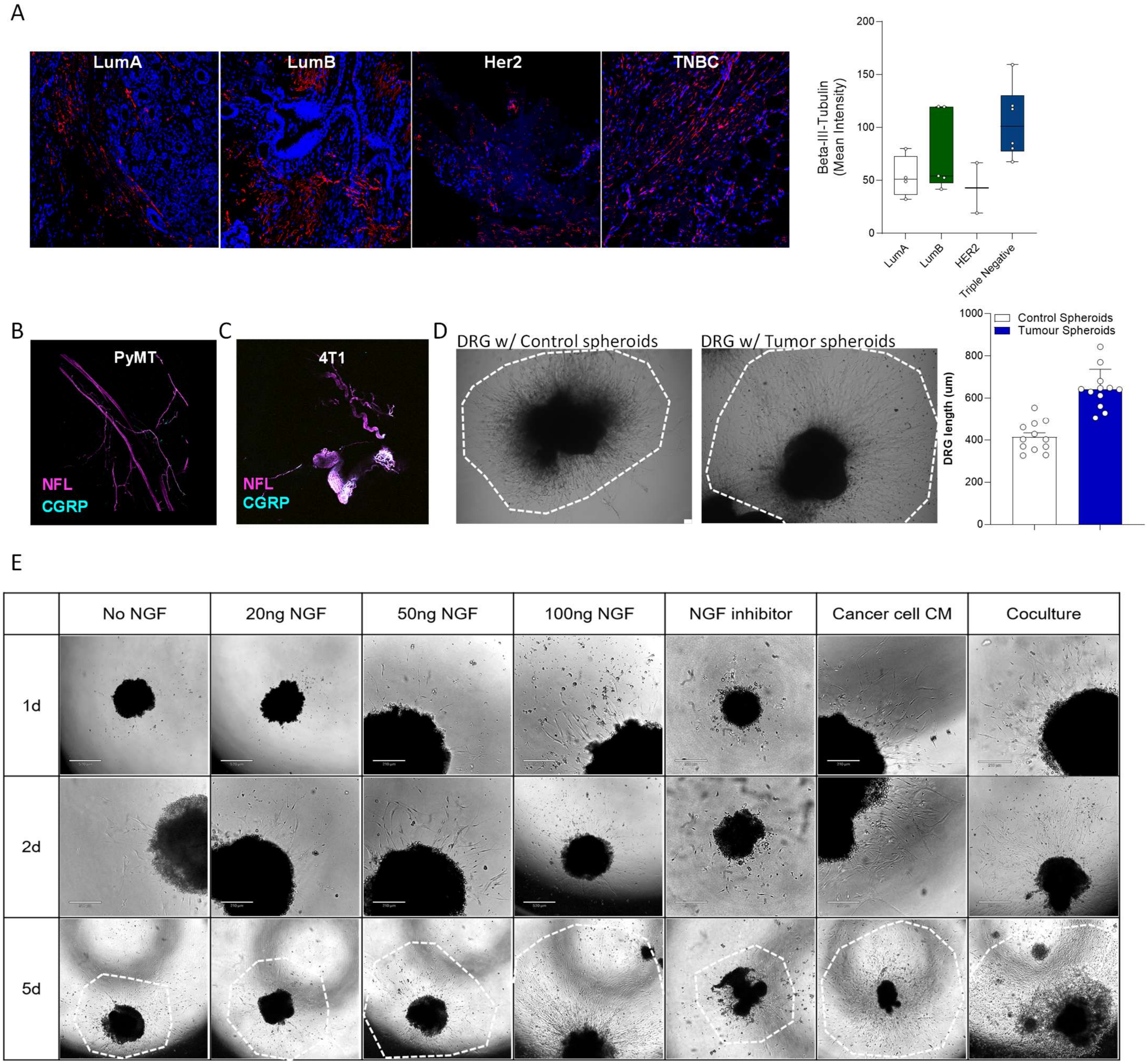

Supplementary Figure 1. Cancer-neuron crosstalk promotes sensory fiber remodeling and neurite outgrowth

A, Representative immunofluorescence images and quantification of beta-III-tubulin staining in LumA, LumB, $HER2^+$ and triple-negative breast cancer patient biopsies.

B,C, Representative immunofluorescence images of EO771 (B), MMTV-PyMT (B) and 4T1 (C) tumors stained for neurofilament light (NFL, magenta) and CGRP (cyan). Merged images show $CGRP^+$ sensory fibers associated with neurofilament-labeled structures across breast tumor models.

D, Representative brightfield images and quantification of DRG neurite extension after co-culture with control mammary gland spheroids or tumor spheroids for 10 days. n = 12; scale bar, 250 μm.

E, Representative time-course images of DRG neurite growth under no NGF, increasing NGF concentrations, the NGF inhibitor Ro 08-2750 (1 μM; selectively inhibits NGF binding to p75NTR over TRKA), cancer-cell conditioned medium, or cancer-cell co-culture at days 1, 2 and 5. Tumor-derived and NGF-associated cues promote sensory neurite remodeling.

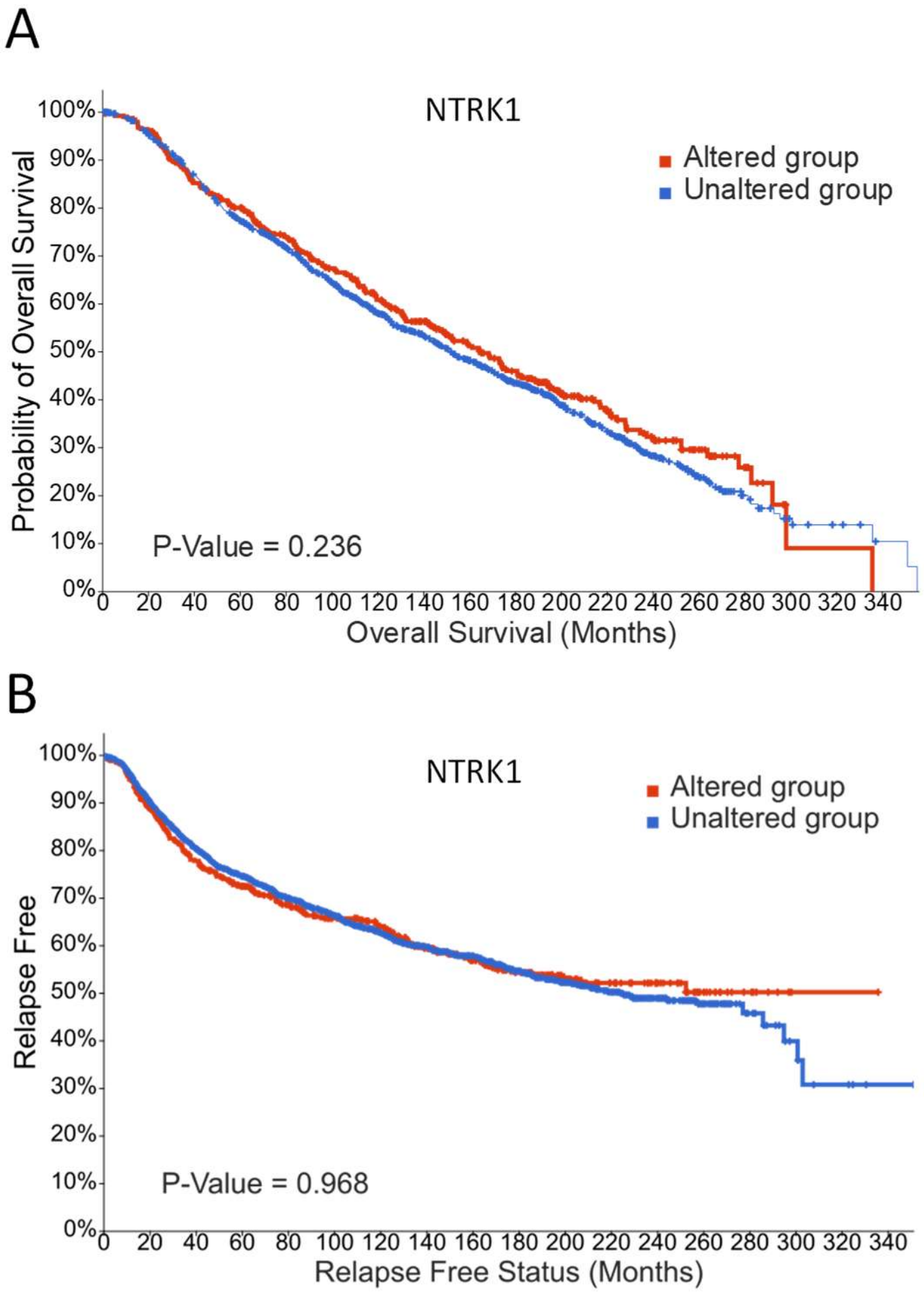

Supplementary Figure 2. NTRK1 alterations are not associated with breast cancer patient survival

A,B, Analysis of the NGF-TrkA/p75NTR axis in the METABRIC breast cancer cohort shows that NTRK1 alterations are not associated with overall survival (A) or relapse-free survival (B).

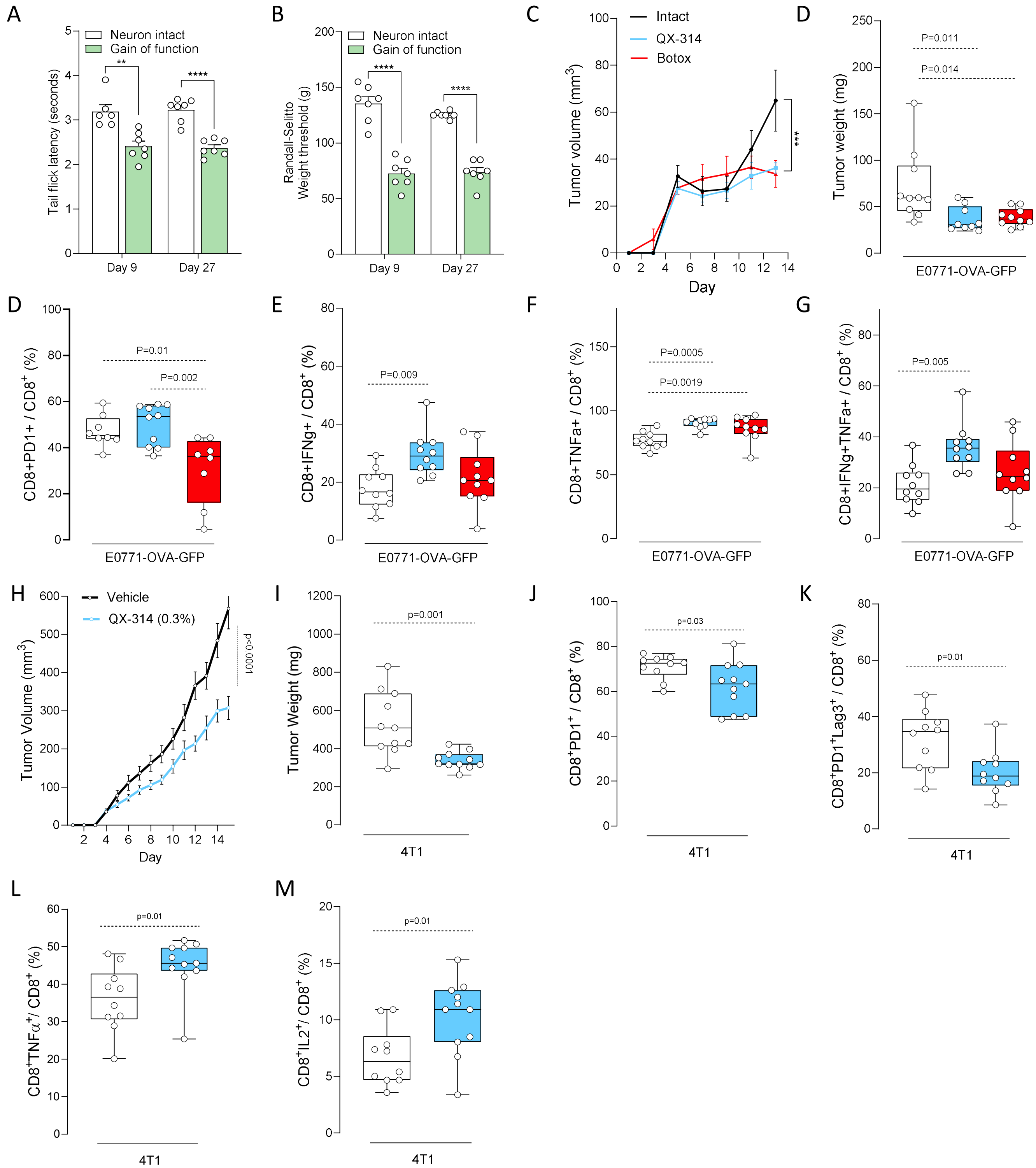
A
Neuron intact
Gain of function
Tail flick latency (seconds)
Day 9
Day 27
**
****
B
Neuron intact
Gain of function
Randall-Selitto
Weight threshold (g)
Day 9
Day 27
****
****
C
Intact
QX-314
Botox
Tumor volume (mm3)
Day
***
D
Tumor weight (mg)
P=0.011
P=0.014
E0771-OVA-GFP
D
CD8+PD1+ / CD8+ (%)
P=0.01
P=0.002
E0771-OVA-GFP
E
CD8+IFNg+ / CD8+ (%)
P=0.009
E0771-OVA-GFP
F
CD8+TNFa+ / CD8+ (%)
P=0.0005
P=0.0019
E0771-OVA-GFP
G
CD8+IFNg+TNFa+ / CD8+ (%)
P=0.005
E0771-OVA-GFP
H
Vehicle
QX-314 (0.3%)
Tumor Volume (mm3)
Day
p<0.0001
I
Tumor Weight (mg)
p=0.001
4T1
J
CD8+PD1+ / CD8+ (%)
p=0.03
4T1
K
CD8+PD1+Lag3+ / CD8+ (%)
p=0.01
4T1
L
CD8+TNFα+/ CD8+ (%)
p=0.01
4T1
M
CD8+IL2+/ CD8+ (%)
p=0.01
4T1

Supplementary Figure 3. Pharmacological modulation of sensory neuron activity alters tumor growth and enhances CD8⁺ T cell effector function

A,B, Behavioral validation of sensory neuron modulation. Tail-flick latency is reduced in gain-of-function mice compared with neuron-intact controls at days 9 and 27, indicating increased sensory neuron activity (A). The Randall-Selitto assay shows decreased mechanical withdrawal thresholds in gain-of-function mice, confirming enhanced nociceptive sensitivity (B). Statistical significance was assessed using an unpaired t-test. Data are presented as mean ± SEM; n = 6-7.

C,D, Tumor growth following pharmacological inhibition of sensory neuron activity. EO771 cells (1×10^6) were orthotopically implanted into the mammary fat pad of wild-type mice. Mice were treated with vehicle control, QX-314 ([AUTHOR QUERY: add dose]) or botulinum toxin ([AUTHOR QUERY: add dose]). Tumor growth curves show reduced tumor volume in QX-314- and botulinum toxin-treated mice compared with vehicle-treated controls (C). Endpoint tumor weight was significantly decreased following QX-314 or botulinum toxin treatment (D). Statistical significance was assessed using two-way ANOVA for tumor growth and an unpaired t-test for endpoint weight. Data are presented as mean ± SEM; n = 10.

E-G, Flow cytometric analysis of tumor-infiltrating $CD8^+$ T cells in EO771 tumors. The frequency of $PD\text{-}1^+$ cells within the $CD8^+$ T cell population is reduced in botulinum toxin-treated mice compared with control and QX-314-treated mice (E). The percentage of $IFN\gamma^+$ $CD8^+$ T cells is increased in QX-314-treated mice (F). Frequencies of $TNF\alpha^+$ and $IFN\gamma^+TNF\alpha^+$ $CD8^+$ T cells are increased following QX-314 and/or botulinum toxin treatment, indicating enhanced $CD8^+$ T cell effector function (G). Statistical significance was assessed using an unpaired t-test. Data are presented as mean ± SEM; n = 10.

H,I, Tumor growth in the 4T1 breast cancer model. Following orthotopic injection of 4T1 cells (5×10^5), wild-type mice were treated with QX-314 (0.3%) or vehicle control. QX-314 treatment reduced tumor volume over time (H), and endpoint tumor weight was decreased following QX-314 treatment (I). Statistical significance was assessed using two-way ANOVA for tumor growth and an unpaired t-test for endpoint weight. Data are presented as mean ± SEM; n = 12.

J-M, Flow cytometric analysis of $CD8^+$ T cell phenotype in 4T1 tumors. The frequency of $PD\text{-}1^+$ $CD8^+$ T cells is reduced in QX-314-treated mice (J). The frequency of $PD\text{-}1^+LAG3^+$ exhausted $CD8^+$ T cells is reduced in QX-314-treated tumors (K). $TNF\alpha^+$ $CD8^+$ T cells are increased following QX-314 treatment (L). $IL\text{-}2^+$ $CD8^+$ T cells are increased in QX-314-treated mice, consistent with enhanced T cell functionality (M). Statistical significance was assessed using an unpaired t-test. Data are presented as mean ± SEM; n = 10-11.

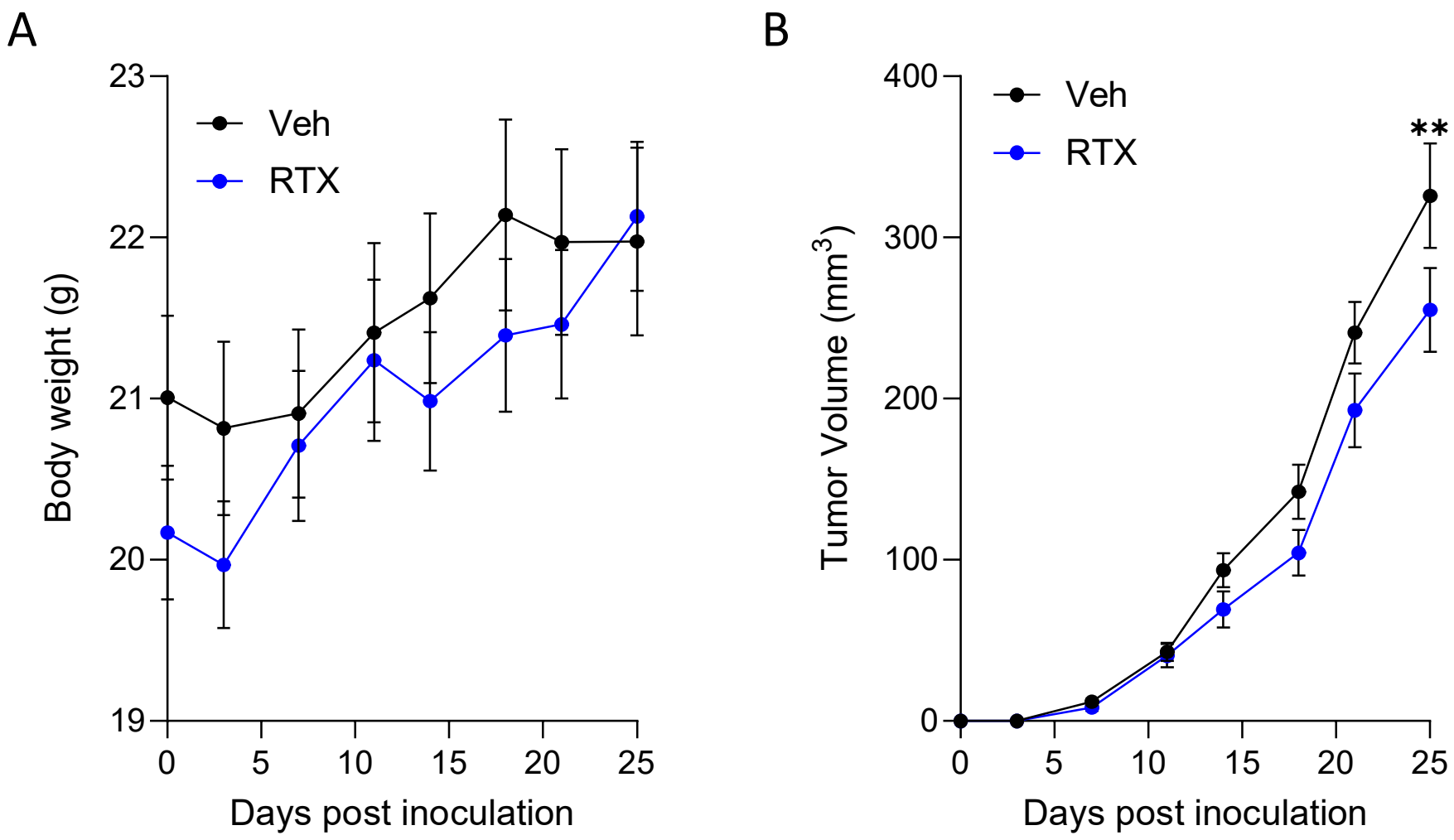

Supplementary Figure 4. RTX ablation suppresses 4T1 tumor growth

A, Body weight of vehicle- (Veh) and RTX-treated mice following orthotopic inoculation of 4T1 cells.
B, Tumor growth curves of vehicle- and RTX-treated mice after 4T1 inoculation. RTX treatment reduced tumor growth compared with vehicle-treated controls. Data are presented as mean ± SEM; n = 13. Statistical significance was determined by two-way ANOVA with multiple-comparisons test. **$p < 0.01$.

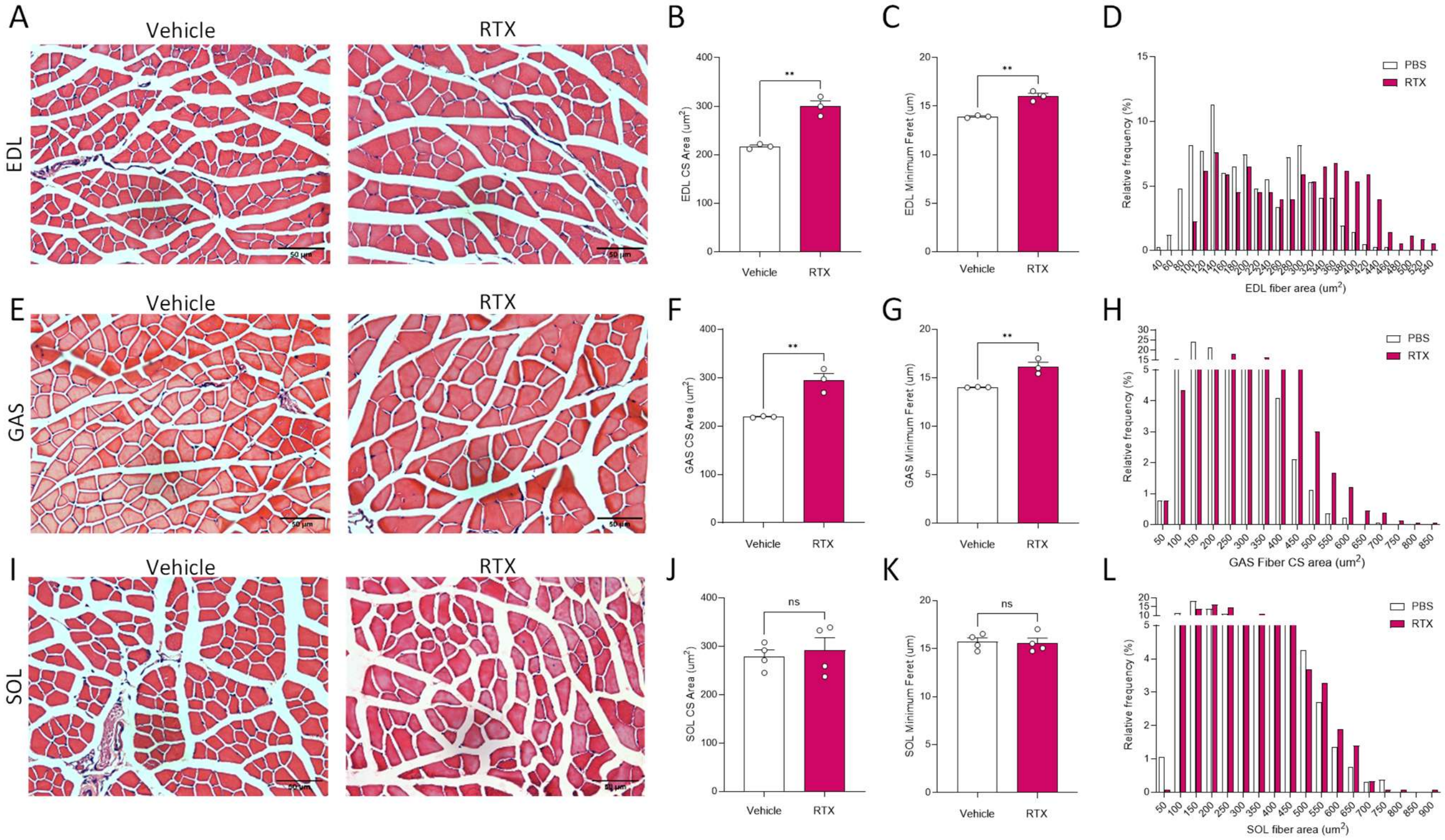

Supplementary Figure 5. Sensory nerve ablation induces skeletal muscle hypertrophy

A-L, Chemical depletion of sensory nerves significantly increases skeletal muscle fiber cross-sectional area compared with controls. Representative images of extensor digitorum longus muscle are shown in A. Fiber area and minimum Feret diameter are significantly increased following sensory nerve depletion in EDL muscle (B-D) and gastrocnemius (GAS) muscle (E-H), but not in soleus (SOL) muscle (I-L). Groups were compared using an unpaired t-test or a Mann-Whitney test when data were not normally distributed.

Data are presented as mean ± SEM. *$p < 0.05$, **$p < 0.01$.

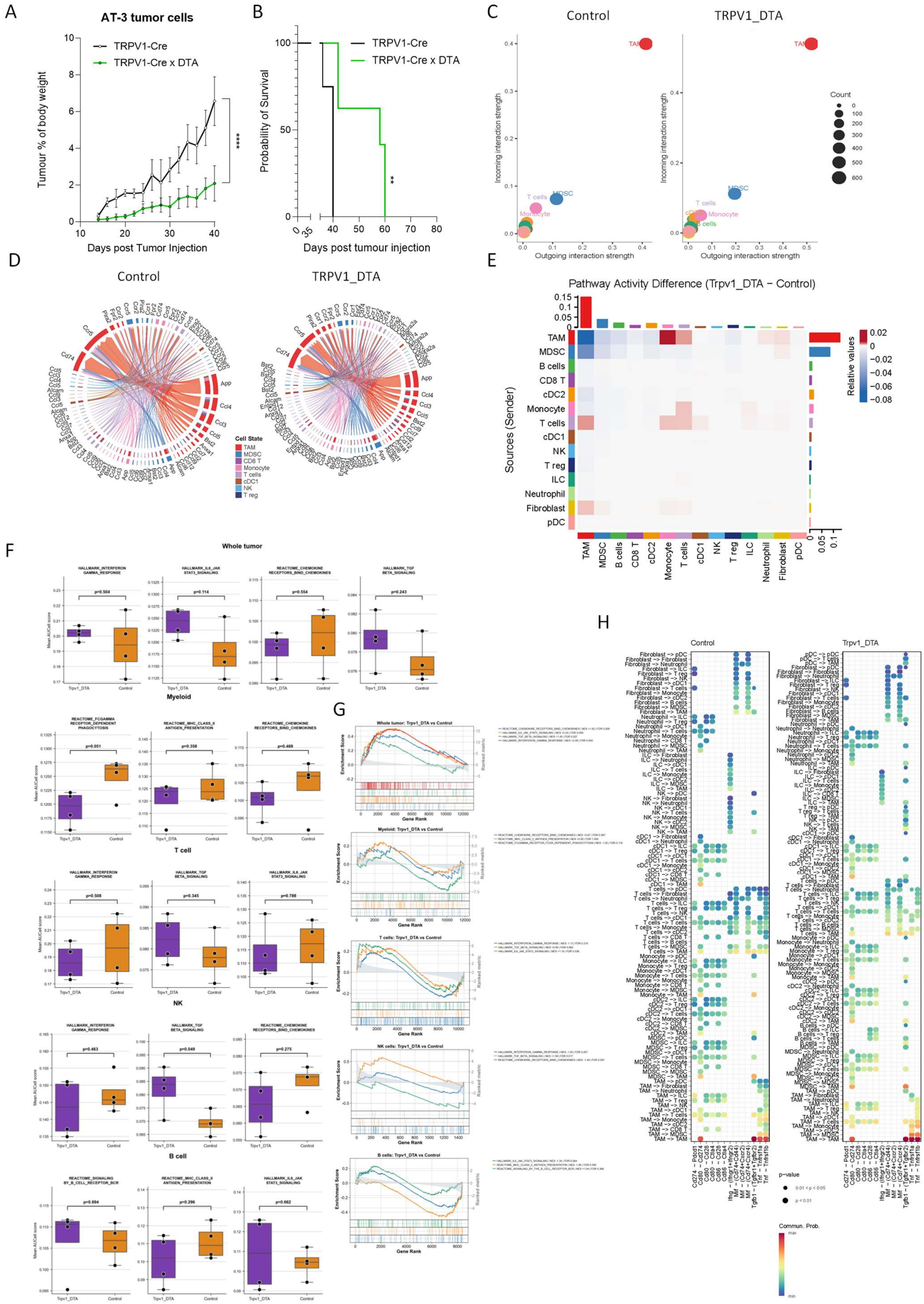
A
AT-3 tumor cells
TRPV1-Cre
TRPV1-Cre x DTA
Tumour % of body weight
Days post Tumor Injection
****
B
TRPV1-Cre
TRPV1-Cre x DTA
Probability of Survival
Days post tumour injection
**
C
Control
TRPV1_DTA
Incoming interaction strength
Outgoing interaction strength
TAM
MDSC
T cells
Monocyte
B cells
Count
D
Control
TRPV1_DTA
Cell State
TAM
MDSC
CD8 T
Monocyte
T cells
cDC1
NK
T reg
E
Pathway Activity Difference (Trpv1_DTA − Control)
Sources (Sender)
Relative values
TAM
MDSC
B cells
CD8 T
cDC2
Monocyte
T cells
cDC1
NK
T reg
ILC
Neutrophil
Fibroblast
pDC
F
Whole tumor
Myeloid
T cell
NK
B cell
Mean AUCell score
Trpv1_DTA
Control
G
Whole tumor: Trpv1_DTA vs Control
Myeloid: Trpv1_DTA vs Control
T cells: Trpv1_DTA vs Control
NK cells: Trpv1_DTA vs Control
B cells: Trpv1_DTA vs Control
Enrichment Score
Ranked metric
Gene Rank
H
Control
Trpv1_DTA
p-value
Commun. Prob.

Supplementary Figure 6. Loss of TRPV1⁺ neurons reshapes intercellular signaling in the tumor microenvironment

A,B, AT-3 tumor cells (1×10^6) were orthotopically implanted into the mammary fat pad. Tumor growth was significantly reduced in Trpv1-Cre::DTA^fl/wt mice compared with Trpv1^wt::DTA^fl/wt controls (A). Kaplan-Meier analysis shows survival in TRPV1 neuron-ablated mice and controls (B). n = 5 per group. Paired t-test (A) and Mantel-Cox test (B). Data are presented as mean ± SEM.

C-E, EO771 cells (2×10^5) were orthotopically injected into the fourth mammary fat pad of 8-week-old female nociceptor-intact (Trpv1^wt::DTA^fl/wt) and nociceptor-ablated (Trpv1-Cre::DTA^fl/wt) mice. Single-cell RNA sequencing was used to analyze tumor-infiltrating immune cells and intercellular communication. CellChat analysis of ligand-receptor interactions shows incoming interaction strength across immune populations in control and Trpv1-Cre::DTA^fl/wt tumors, with prominent signaling activity in tumor-associated macrophages (TAMs; C). Chord plots show the top 10 signaling pathways among key immune cell types in control and Trpv1-Cre::DTA^fl/wt tumors (D). A pathway-level heatmap compares signaling strength between groups, with increased signaling in Trpv1-Cre::DTA^fl/wt tumors highlighted (E).

F,G, Pseudobulk pathway analyses comparing control and Trpv1-Cre::DTA^fl/wt tumors across whole immune, myeloid, T cell, NK-cell and B-cell compartments. Box plots summarize module-score differences for selected pathways (F), and enrichment plots show representative gene-set enrichment analyses (G), highlighting enhanced dendritic-cell, T cell and myeloid activation programs after TRPV1-lineage neuron ablation.

H, Bubble plots comparing signaling activity of selected immunoregulatory pathways between groups. Dot size indicates interaction strength, and color indicates statistical significance. Enhanced signaling is observed in pathways involving TAM-TAM interactions and key mediators, including CD80, TGFB1, TNFRSF1A and TNFRSF1B, indicating altered immunoregulatory and inflammatory communication following sensory neuron ablation.

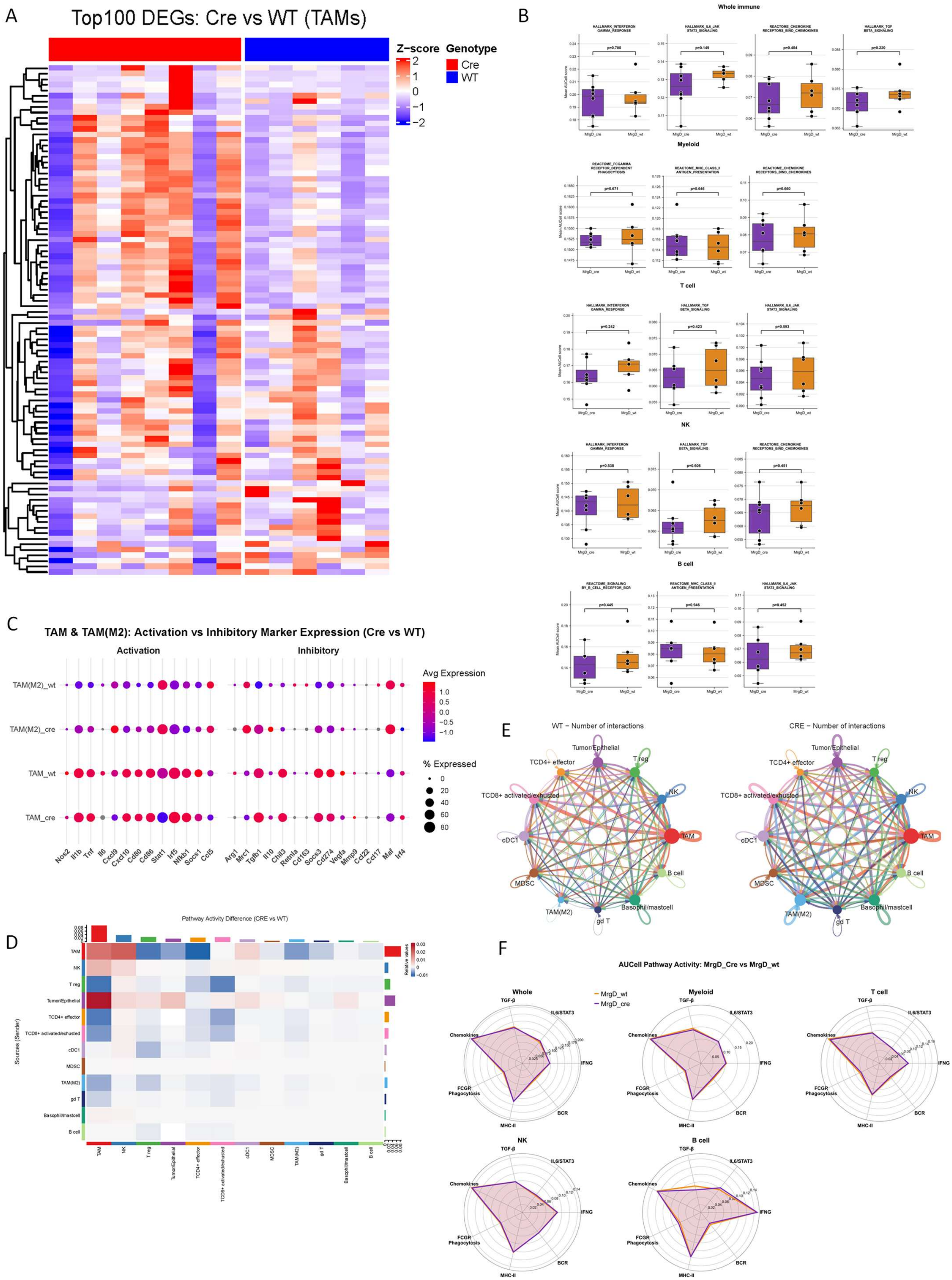
A
Top100 DEGs: Cre vs WT (TAMs)
Z-score
Genotype
Cre
WT
B
Whole immune
HALLMARK_INTERFERON GAMMA_RESPONSE
HALLMARK_IL6_JAK STAT3_SIGNALING
REACTOME_CHEMOKINE RECEPTORS_BIND_CHEMOKINES
HALLMARK_TGF BETA_SIGNALING
p=0.700
p=0.149
p=0.484
p=0.220
Mean AUCell score
MrgD_cre
MrgD_wt
Myeloid
REACTOME_FCGAMMA RECEPTOR_DEPENDENT PHAGOCYTOSIS
REACTOME_MHC_CLASS_II ANTIGEN_PRESENTATION
REACTOME_CHEMOKINE RECEPTORS_BIND_CHEMOKINES
p=0.671
p=0.646
p=0.660
T cell
HALLMARK_INTERFERON GAMMA_RESPONSE
HALLMARK_TGF BETA_SIGNALING
HALLMARK_IL6_JAK STAT3_SIGNALING
p=0.242
p=0.423
p=0.593
NK
HALLMARK_INTERFERON GAMMA_RESPONSE
HALLMARK_TGF BETA_SIGNALING
REACTOME_CHEMOKINE RECEPTORS_BIND_CHEMOKINES
p=0.538
p=0.608
p=0.451
B cell
REACTOME_SIGNALING BY_B_CELL_RECEPTOR_BCR
REACTOME_MHC_CLASS_II ANTIGEN_PRESENTATION
HALLMARK_IL6_JAK STAT3_SIGNALING
p=0.445
p=0.946
p=0.452
C
TAM & TAM(M2): Activation vs Inhibitory Marker Expression (Cre vs WT)
Activation
Inhibitory
TAM(M2)_wt
TAM(M2)_cre
TAM_wt
TAM_cre
Nos2
Il1b
Tnf
Il6
Cxcl9
Cxcl10
Cd80
Cd86
Stat1
Irf5
Nfkb1
Socs1
Ccl5
Arg1
Mrc1
Tgfb1
Il10
Chil3
Retnla
Cd163
Socs3
Cd274
Vegfa
Mmp9
Ccl22
Ccl17
Maf
Irf4
Avg Expression
% Expressed
D
Pathway Activity Difference (CRE vs WT)
Sources (Sender)
Relative values
TAM
NK
T reg
Tumor/Epithelial
TCD4+ effector
TCD8+ activated/exhusted
cDC1
MDSC
TAM(M2)
gd T
Basophil/mastcell
B cell
E
WT − Number of interactions
CRE − Number of interactions
Tumor/Epithelial
T reg
NK
TAM
B cell
Basophil/mastcell
gd T
TAM(M2)
MDSC
cDC1
TCD8+ activated/exhausted
TCD4+ effector
F
AUCell Pathway Activity: MrgD_Cre vs MrgD_wt
MrgD_wt
MrgD_cre
Whole
Myeloid
T cell
NK
B cell
TGF-β
IL6/STAT3
IFNG
BCR
MHC-II
FCGR Phagocytosis
Chemokines

Supplementary Figure 7. MrgD$^+$ sensory neuron ablation reprograms tumor-associated macrophage activation and communication networks

A, Heatmap showing the top 100 differentially expressed genes (DEGs) in tumor-associated macrophages (TAMs) from MrgD-Cre mice compared with wild-type controls. The heatmap illustrates distinct TAM transcriptional profiles between genotypes.

B, Pseudobulk pathway-score comparisons across whole immune, myeloid, T cell, NK-cell and B-cell compartments in MrgD-Cre and wild-type tumors.

C, Bubble plot comparing activation and inhibitory marker expression in TAM and TAM(M2)-like populations from MrgD-Cre and wild-type mice, highlighting shifts in macrophage polarization states.

D, Heatmap of pathway-level signaling strength comparing MrgD-Cre and wild-type tumors. Each value represents the relative change in communication weight for a given pathway, with increased signaling activity prominently observed in TAM populations.

E, Circle plots summarizing global cell-cell communication networks. Node size reflects total signaling activity for each cell type, whereas edge width indicates the number of interactions, revealing genotype-dependent differences in intercellular communication.

F, AUCell pathway-activity radar plots summarize pathway activity across immune compartments, highlighting genotype-dependent remodeling of inflammatory, chemokine, interferon, BCR, MHC-II and TGFβ-related programs.

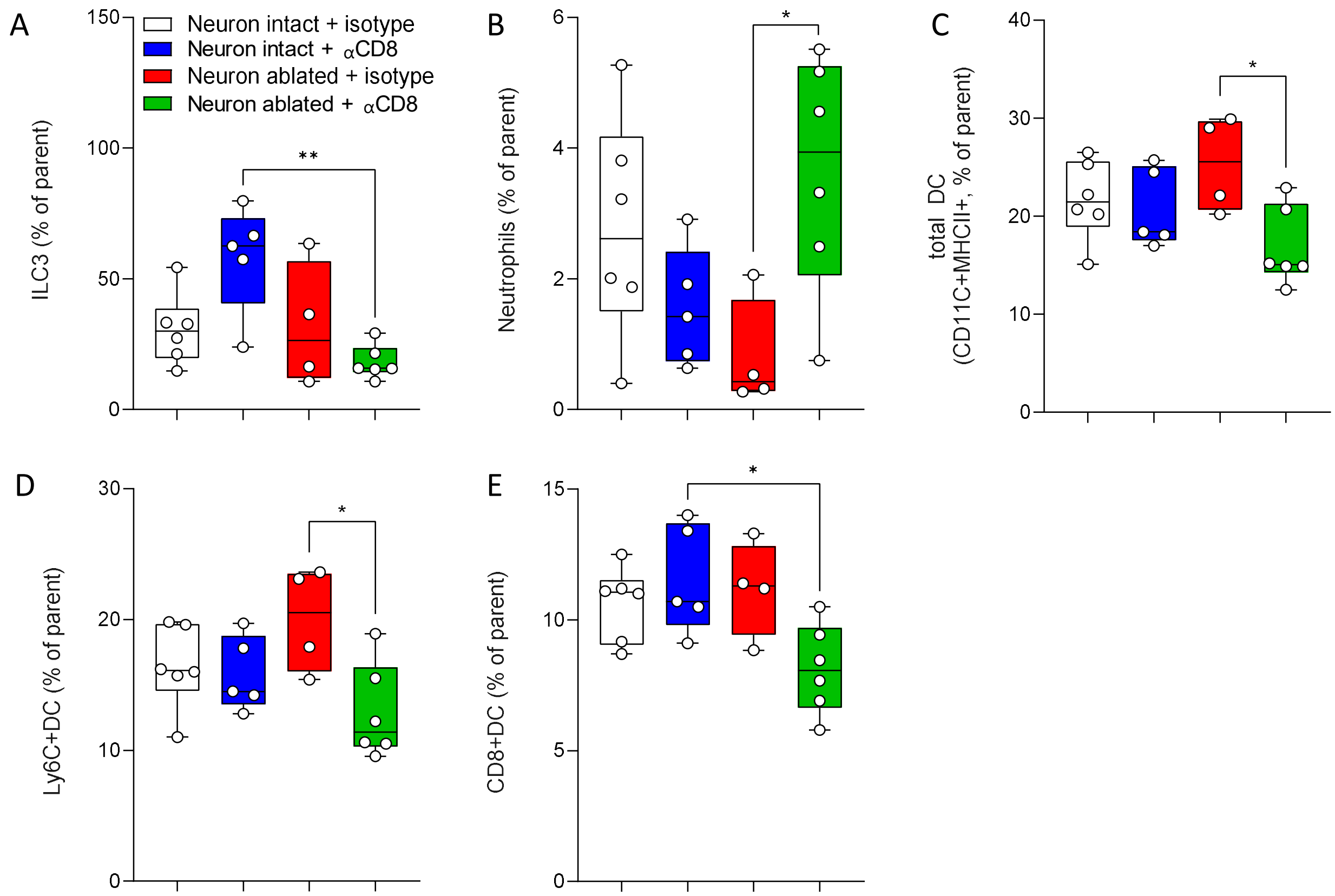

Supplementary Figure 8. Sensory neurons and CD8^{+} T cells regulate innate immune composition in tumors

A-E, Flow cytometric analysis of tumor-infiltrating innate immune cell populations in neuron-intact and neuron-ablated mice, with or without CD8^{+} T cell depletion. Mice were treated with isotype control or anti-CD8 antibody under neuron-intact or neuron-ablated conditions. ILC3 frequency, expressed as a percentage of the parent population, is reduced in neuron-ablated, CD8-depleted mice (green) compared with neuron-intact, CD8-depleted mice (blue; A). Neutrophil frequency is increased in neuron-ablated, CD8-depleted mice (green) compared with neuron-ablated isotype controls (red; B). Total dendritic cells (DCs; CD11c^{+}MHCII^{+}) are decreased in neuron-ablated, CD8-depleted mice (green) compared with neuron-ablated controls (red; C). Ly6C^{+} DCs are reduced in neuron-ablated, CD8-depleted mice (green) compared with neuron-ablated controls (red; D). CD8^{+} DCs are decreased in neuron-ablated, CD8-depleted mice (green) compared with neuron-intact, CD8-depleted mice (blue; E).

Statistical significance was assessed using an unpaired t-test. Data are presented as mean ± SEM; n = 4-6.
